# A scalable solution recipe for a Ag-based neuromorphic device

**DOI:** 10.1186/s11671-023-03906-5

**Published:** 2023-10-09

**Authors:** Tejaswini S. Rao, Indrajit Mondal, Bharath Bannur, Giridhar U. Kulkarni

**Affiliations:** Chemistry and Physics of Materials Unit, Jawaharlal Nehru Centre for Advanced Scientific Research, Jakkur P.O., Bangalore, 560064 India

**Keywords:** Self-forming, Dewetting, Chemical process, Hierarchical structures, Neuromorphic device, Associative learning, Scalability

## Abstract

Integration and scalability have posed significant problems in the advancement of brain-inspired intelligent systems. Here, we report a self-formed Ag device fabricated through a chemical dewetting process using an Ag organic precursor, which offers easy processing, scalability, and flexibility to address the above issues to a certain extent. The conditions of spin coating, precursor dilution, and use of solvents were varied to obtain different dewetted structures (broadly classified as bimodal and nearly unimodal). A microscopic study is performed to obtain insight into the dewetting mechanism. The electrical behavior of selected bimodal and nearly unimodal devices is related to the statistical analysis of their microscopic structures. A capacitance model is proposed to relate the threshold voltage (V_th_) obtained electrically to the various microscopic parameters. Synaptic functionalities such as short-term potentiation (STP) and long-term potentiation (LTP) were emulated in a representative nearly unimodal and bimodal device, with the bimodal device showing a better performance. One of the cognitive behaviors, associative learning, was emulated in a bimodal device. Scalability is demonstrated by fabricating more than 1000 devices, with 96% exhibiting switching behavior. A flexible device is also fabricated, demonstrating synaptic functionalities (STP and LTP).

**Supplementary Information:**

The online version contains supplementary material available at 10.1186/s11671-023-03906-5.

## Introduction

There is an increasing demand to achieve tools with a parallel processing ability similar to what the human brain is capable of, and this has caused an upsurge in activities in the area of neuromorphic devices. Such devices are expected to emulate cognitive behaviors but with modest energy consumption. Numerous materials such as the 2D materials [1, 2], nanowire networks [3–5], ferroelectric materials [6, 7], organic materials [8, 9], metals [10, 11], metallic oxides [12–15], and phase change materials [16] have been exploited as active elements to quantitatively emulate the behavioral aspects including short-term potentiation (STP), long-term potentiation (LTP), and classical conditioning. Toward this, varied device architectures based on different mechanisms such as the electrochemical metallization (ECM) [17, 18], valence change (VCM) [19, 20], ion migration [21, 22], charge trapping [23–25], and phase change (PCM) [26, 27] have been incorporated. Among these, ECM-based devices operating with low voltages and currents for nanoscale switching [28, 29] hold good promise. There are a few reports on the emulation of synaptic behaviors solely based on electromigration, too [30, 31].

Electrochemical metallization and electromigration relate to metallic filamentary formation induced by an external electrical stimulus, leading to discrete conductance states [27]. Lutz et al. [32] report an ECM-driven α−Ag_2+δ_S device fabricated with Pt electrodes which have mimicked the amoeba-inspired ‘tug of war’ model. Terabe et al. [33] have demonstrated logic states in the device Ag/Ag_2_S/Pt with a 1 nm spacing between Ag_2_S and Pt. Similar device geometries were followed in studies by Ohno et al. [34] and Barbera et al. [35], while the latter had no spacing between Ag_2_S and Pt. While such approaches are successful in emulating learning behaviors, the fabrication protocols continue to pose challenges toward scalability and integration, which, despite being important, have not been well addressed in the literature. Studies on scalable devices typically involve intricate lithography processes [36, 37] and may not be cost-effective.

In this context, there have been constant efforts in the literature to develop recipes for self-formed nanosystems, thus overcoming the challenges mentioned above. Such systems, as formed, come with structural disorders that are often taken to mimic, though remotely, the biological neural networks. Importantly, disordered systems tend to exhibit nonlinear electrical properties that are essential for mimicking neuromorphic functionalities [38]. The self-forming of the active material can be achieved through either physical or chemical processes. Thus, a self-organized percolating network of Sn nanoparticles deposited by sputtering [39–42] and a nanostructured Ag film obtained through deposition followed by thermal dewetting [43, 44] have been employed as active elements. Although the device fabrication is straightforward, it does involve vacuum processing which is not favored when scalability is an issue at hand. Designing an active material through chemical processes is therefore advantageous for easy processing, scalability, and cost-effectiveness, avoiding expensive lithography techniques as well as vacuum conditions. For example, reservoir computing was performed in random networks of CNTs [45] and Ag–Ag_2_S core–shell nanoparticles [46] obtained via chemical routes. It has also been shown that such self-organized networks of CNTs [47] and nanowires [48–50] bear some resemblance to biological neural systems and exhibit neuromorphic functionalities. However, chemically processed active materials are less explored in regard to the tuning of the self-formed structures and emulating advanced cognitive behaviors.

Encouraged by the earlier results from the laboratory on higher-order cognitive behaviors realized using physically deposited and dewetted Ag films [43, 44] in this study, we undertook to explore the possibility of obtaining similar hierarchical nanostructures via chemical routes. It is noteworthy that the duration for the active material (dewetted Ag) preparation is less than a minute outperforming the lithography-based fabrication processes. Specifically, using a modified Ag organic precursor, conditions of spin coating and thermal annealing were optimized, aiming for the desired nanostructures consisting of separated Ag islands (particles > 0.3 µm^2^—Fig. S1 in the supplementary information (SI)) with nanoparticles filled in between. During optimization, high-resolution optical and scanning electron microscopy (SEM) techniques were employed to gain insight into the film formation and dewetting stages. Using this nanostructure as the active element in a two-terminal geometry, the electrical switching characteristics were obtained for devices with varied Ag fill factor (FF) from ~ 18 to 51%, the corresponding switching voltages decreasing from 70 to as small as 1 V. A capacitance model is also proposed to support the observations. Synaptic functionalities, STP and LTP, have been emulated, and devices with different dewetted patterns have been compared. A device with 45% FF could exhibit higher-order associative learning behavior. Scalability is also demonstrated by fabricating more than 1000 devices on a ~ 10 × 10 cm^2^ substrate in single-step processing. A device was made on a flexible substrate as well, and its STP and LTP behaviors are demonstrated.

## Experimental section

### Device fabrication

A glass slide was cut into 1 × 1 cm^2^ and cleaned via sonication in acetone and IPA for 15 min. After drying under nitrogen purge, the substrates were ozone cleaned for 10 min. The Ag precursor (SC-100, 20 wt%, Kunshan Hisense Electronics Co., Ltd, China) was diluted according to the requirements and spin-coated at the desired RPM (see Table S1 in the SI for more details) on the substrates. Immediately after spin coating, the substrates were annealed at 270 ºC for 30 s on a pre-heated hotplate. The substrates were masked suitably to obtain an active area of ~ 600 × 40 µm^2^. Finally, the devices were fabricated by depositing Au electrodes on the Ag precursor-coated glass substrates using an e-beam evaporator at ∼10^−6^ Torr.

### Characterization

SEM images were captured using Apreo 2 S SEM, Thermo Fischer Scientific. Electrical measurements were carried out using Keithley 2450 SMU. TEM images were captured using JEOL 200 kV. XPS was performed using K-Alpha, Thermofischer and analyzed using the Avantage software. Raman spectra were collected from Xplora plus V1.2 Multiline, Horiba Scientific. XRD was done using Rigaku equipped with a Cu-Kα target of monochromatic wavelength (*λ* = 1.5404 Å). TGA analysis was done using STA600, Perkin Elmer.

## Results and discussion

**Fig. 1:**
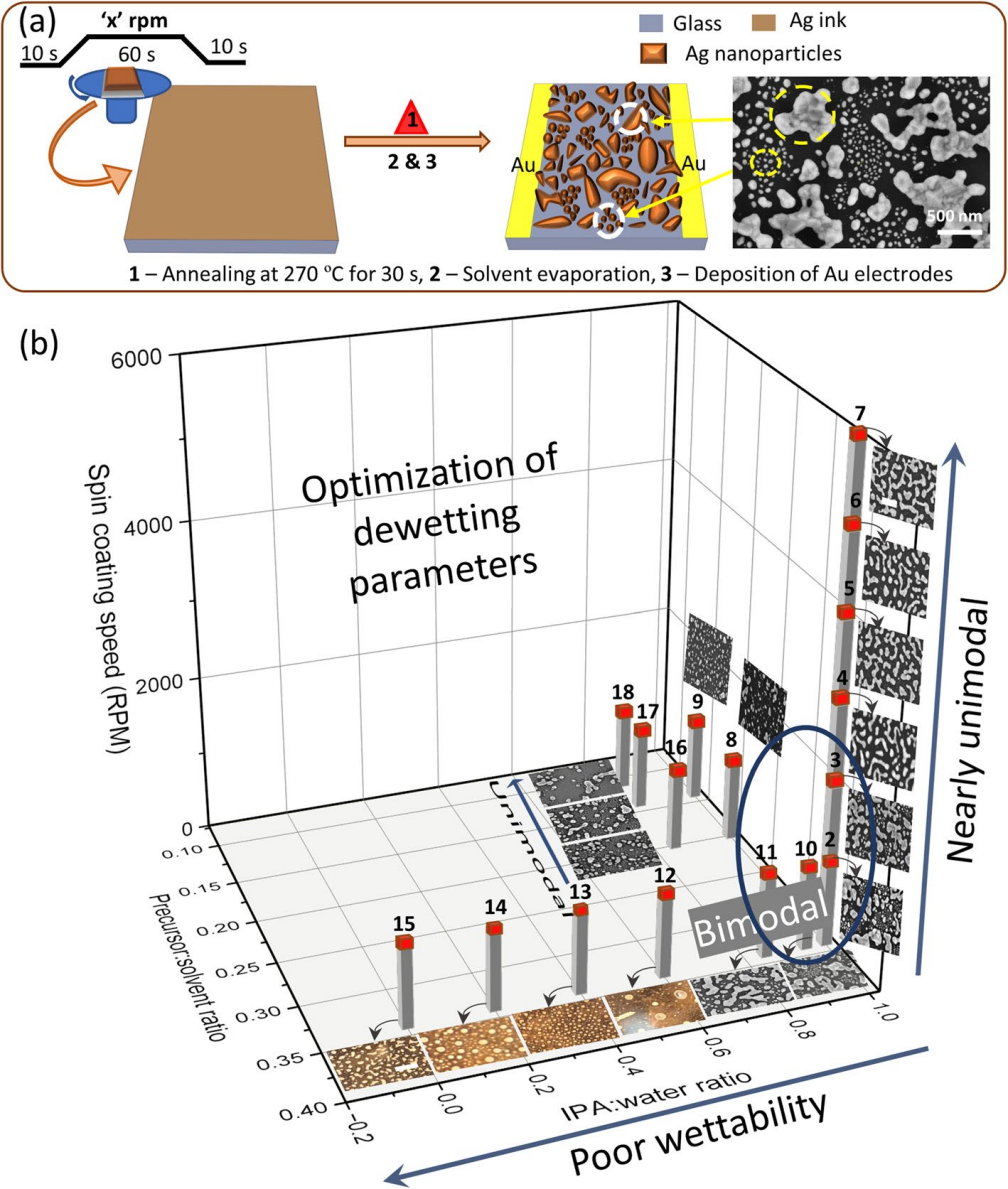
**a** Schematic demonstrating fabrication protocol of the dewetted Ag synaptic device with in-plane Au electrodes. SEM image of a typical dewetted film consisting of a hierarchical network structure is shown on the right. **b** 3D plot showing the variation in dewetting parameters (spin coating speed, precursor: solvent ratio, IPA: water ratio in the solvent) explored to obtain the desired dewetting pattern with the corresponding SEM/optical images displayed adjacent to the data point (red box). The optical images refer to the data points 12 to 15 (Scale bar: 0.1 cm, indicated in image 15), while others are SEM images (Scale bar: 1 µm, indicated in image 7). The scale bar applies to all optical and SEM images

The device fabrication involves only a few steps, schematically described in Fig. 1(a). Briefly, a few drops of modified Ag precursor (see experimental section) were spin-coated onto a 1 × 1 cm^2^ glass substrate which was immediately transferred onto a hotplate at 270 °C and kept for 30 s. This rapid heating caused solvent evaporation followed by decomposition of the organics in the precursor giving rise to metallic Ag films. However, as shown in the SEM image (Fig. 1(a), right), the film was not continuous but appeared dewetted, consisting of agglomerated Ag islands (particles > 0.3 µm^2^) separated by relatively smaller discrete Ag nanoparticles. Statistically, the dewetting pattern is uniform throughout, as evident from the SEM images in different regions of the substrate (Fig. S2). Notably, the dewetting temperature is not high enough to cause any detectable oxidation, as the X-ray diffraction pattern contained peaks corresponding only to Ag metal (Fig. S3(a)). The Ag 3d_5/2_ core-level spectrum was symmetric, with the peak position at ~ 368.2 eV [51], ruling out any significant surface oxidation (Fig. S3(b)).

As already mentioned, disordered structures with nonlinear properties closely resembling the biological neural system are necessary for the emulation of higher-order neuromorphic functionalities. Accordingly, an ideal system expected to mimic the neuromorphic functionalities should host at least two to three orders of hierarchy (particles of different sizes), anisotropic structures and features with sharp curvatures. To obtain the desired dewetting pattern, a number of dewetting patterns were created by varying the spin coating conditions (rpm), precursor:solvent ratio (PSR), and IPA:water ratio in the solvent while keeping the annealing temperature (270 °C) and time (30 s) constant (Fig. 1(b)). Each time, a single parameter was varied while the other two were held constant. Interestingly, a bimodal dewetted pattern consisting of both islands and particles was obtained at 1000 rpm (data point 2, see Fig. 1(b) for all the data points), beyond which the bimodality reduced (data point 3) and reached nearly unimodal distribution with further increment in rpm (data point 4, 5, 6, and 7). In a subsequent optimization for a fixed rpm of 1000, when the PSR was varied, it mostly produced nearly unimodal distribution (only nanoparticles) below a PSR of 0.25 (data points 8, 9, 16, 17, and 18). Next, when the solvent ratio was varied from only IPA (data point 2) to increasing water content (data points 10 to 15), the wettability of the diluted precursor greatly decreased, as seen from the optical images. Thus, only 10 and 11 showed good wettability, whereas 12 to 15 gave non-uniform films. Also, 10 and 11 showed bimodal distributions in the dewetted patterns. Further details of the optimization parameters, magnified SEM, and optical images can be found in Table S1 and Fig. S4, respectively. In summary, data points 2, 3, 10, and 11 exhibited bimodal dewetted patterns, whereas other conditions resulted in either nearly unimodal patterns (4 to 9, 16 to 18) or, simply, non-uniform films (12 to 15). It appears that for bimodal dewetting, besides good wettability, the IPA:water ratio should be higher (> 0.8), the spin coating speed should be low (< 3000 rpm), and the precursor:solvent ratio should not be too low (say > 0.3).

**Fig. 2:**
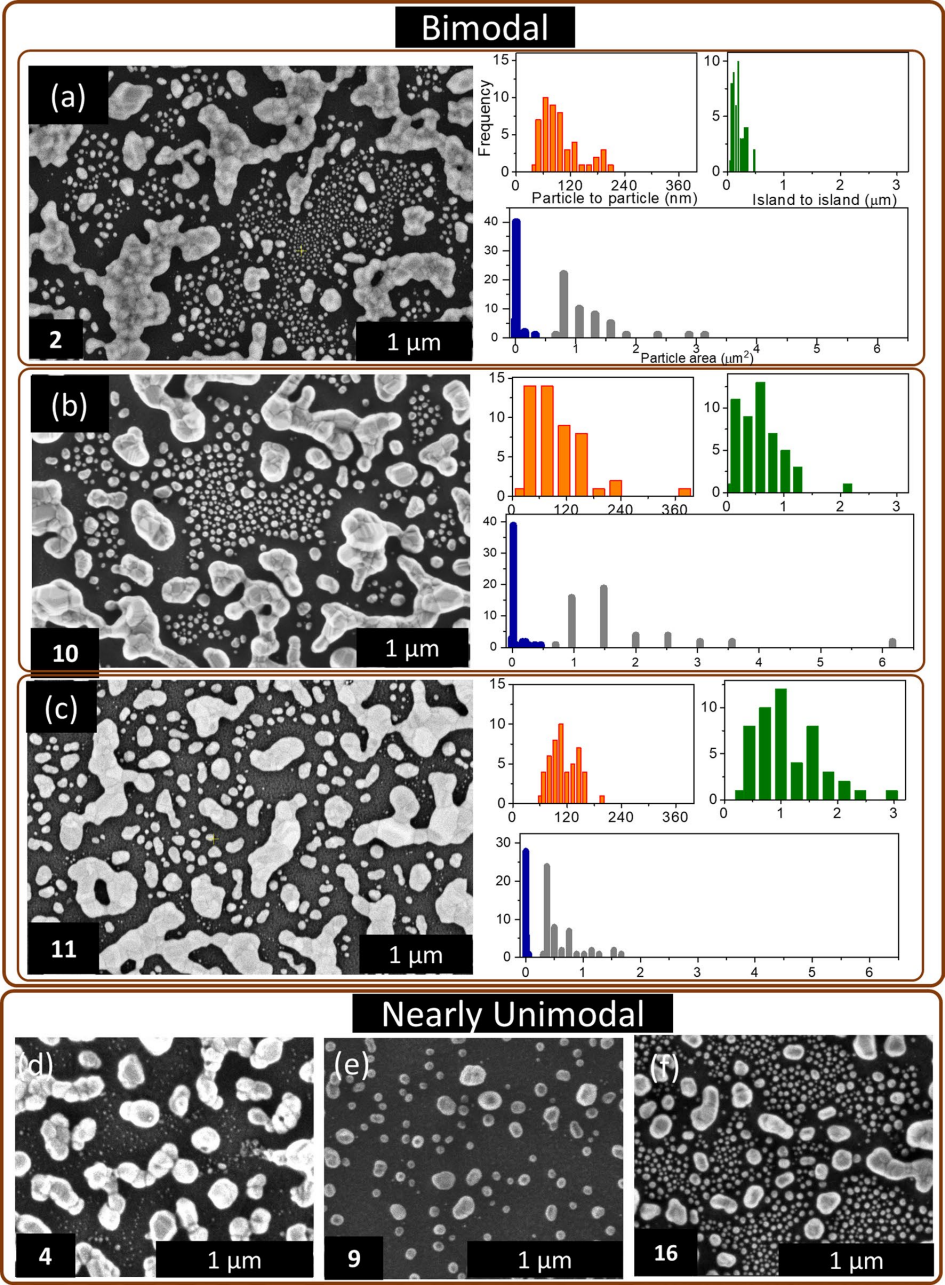
**a–c** SEM images of samples 2, 10, and 11 exhibiting the bimodal distribution and the histograms (adjacent) corresponding to the particle to particle distance, island to island distance, particle area (blue), and island area (gray) of the dewetted patterns. **d–f** SEM images of samples 4, 9, and 16 exhibiting nearly unimodal distributions. However, some distribution in the particle size is seen

Further, to understand the statistical distribution of the islands and particles in the bimodal dewetted patterns, samples 2, 10, and 11 were studied in detail (Fig. 2(a–c)). The histograms adjacent to the SEM images clearly explain the spread of islands and particles. In all three cases, the histograms indicate that the particle to particle distance (orange) varies from 30 to 240 nm with an average value of ~ 100 nm, and the average island to island distance (green) is 0.2, 0.5, and 1.1 µm. However, the Ag nanostructures majorly show two size distributions with particle areas (blue) around 0.02, 0.007, and 0.01 µm^2^ while the average island areas are 1.1, 1.5, and 0.5 µm^2^, respectively (see histograms in gray), in samples 2, 10 and 11, respectively indicating bimodality. All other samples showed nearly unimodal distributions, as observed previously. It may be noted that the terminology used for describing them as ‘nearly unimodal’ is only relative. Here, three representative samples (Figs. 2(d–f)) differing in the particle distributions were considered for the sake of comparing their device behavior, as detailed in a later section. The histograms, indeed, show differences in the particle to particle distance and the particle area (Fig. S5(a–c)). The particle area is quite small (~ 0.01 µm^2^) in samples 16 and 9, whereas it varies up to 0.15 µm^2^ with an average of 0.04 µm^2^ in sample 4. The particle to particle distance is narrowly spread with an average of 50 and 96 nm in samples 16 and 4, respectively. However, sample 9 shows a wide variation with an average of 106 nm. The samples with nearly unimodal distribution showed variation in the particle to particle distance and particle area, whereas, in the samples having bimodal distribution, the variation in island to island distance and island area was also prevalent.

**Fig. 3:**
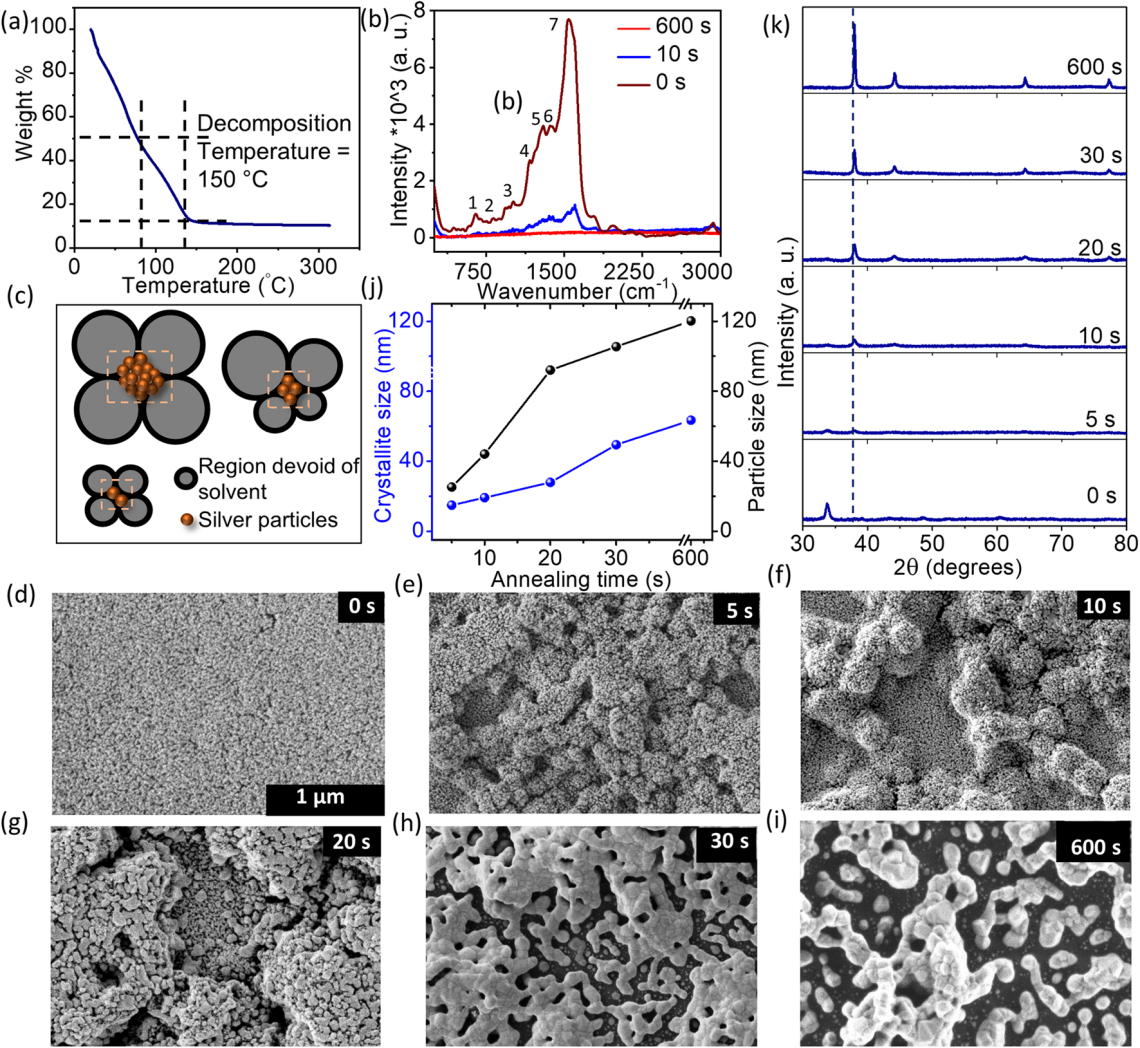
**a** Thermogravimetric analysis of the Ag ink (scan rate: 10 °C/min). **b** Raman spectra show a decline in organic constituents with increasing annealing time. The bands at 648, 934, 1014, 1168 cm^−1^ (bands 1–4) correspond to the stretching vibrations of C–S–C, C–O–C, C–C, and C=S, respectively. The other bands at 1297, 1366, 1542 cm^−1^ (bands 5–7) arise due to the symmetric and anti–symmetric C=O stretching of the carboxylic group, respectively [52, 53]. **c** Schematic showing the dewetting process where the growth of regions devoid of solvent (called holes) due to evaporation leads to a closely packed structure consisting of Ag particles. **d–i** SEM images of the Ag film annealed at ~ 190 °C for different time durations to probe the evolution of dewetted structures (Scale bar: 1 µm). **j** Time-variation of crystallite size and particle size with annealing, as obtained from the XRD pattern and SEM images, respectively. The corresponding XRD patterns are given in **k**. The peaks match the face-centered cubic (fcc) structure of Ag (JCPDS:04–0783)

The dewetting process was also systematically studied by varying the annealing time from 5 to 90 s (SEM images are shown in Fig. S6) while using other synthetic conditions pertaining to sample 11 as an example. The particle to particle distance was found to vary from ~ 70 to 152 nm, while between the islands, from ~ 0.6 to 1.4 µm^2^, both showing roughly an incremental trend with the annealing time (Fig. S7(a–b)). The areas occupied by the individual particles and islands do not seem to have a clear dependence on the annealing time, being in the range of 0.002 to 0.005 and 0.5 to 1.8 µm^2^, respectively (Fig. S7(c–d)). The above analysis of the distribution of particles and islands will be recalled later while explaining the device behavior. However, the annealing temperature of 270 ºC proved to be too fast to capture the early stages of metallization and dewetting. For the purpose of monitoring the evolution of the film morphology, the same Ag precursor was utilized but a lower annealing temperature of 190 °C was chosen such that the metallization of precursor film was still possible while keeping the kinetics slow, tens of seconds to minutes. Indeed, the Ag ink is found to undergo a weight loss of 88% at a temperature of ~ 150 °C, beyond which no observable weight loss is seen, marking complete metallization (Fig. 3(a)).

The decomposition of Ag ink was also confirmed by Raman spectroscopy (Fig. 3(b)). With annealing, the band intensities diminish, indicating the decomposition of the Ag ink accompanied by the loss of the organic constituents. The HRTEM image and the associated SAED pattern of the 600 s annealed Ag film also confirm the formation of metallic Ag (Fig. S8). A quantitative estimation of the increment in Ag content was obtained from the X-ray photoelectron core-level spectra (Fig. S9). As observed, the Ag/C atomic percentage ratio increased from ~ 1.1 to 2.4 with increasing annealing time (0 to 600 s), which clearly implies that the organic constituents diminished with the annealing time. The observed C species must be surface bound, which, however, diminishes at higher annealing temperatures (Fig. S9). We resorted to microscopy data to further understand the dewetting process. In situ optical microscopy imaging ( Figs. S10, S11, and video S1) showed that as the annealing time progressed, the continuous film started appearing grainy with a change in color from green to brown. The emergence of holes and denser regions hold enough clues for the eventual formation of disconnected particles. This observation is in line with the literature reports, which hold that the dewetting occurs through nucleation and growth of particles facilitated by the hole formation (regions devoid of solvent) due to solvent evaporation [54, 55] (Fig. 3(c)).

From the SEM images, it is evident that the as-coated Ag precursor film (0 s, Fig. 3(d)) is nearly uniform with no distinguishable features. However, after 5 s annealing (Fig. 3(e)), the film appears grainier implying the onset of nucleation of particles due to the decomposition and Ag agglomeration. With further annealing, nucleation and growth become apparent, causing the Ag particles to grow bigger (Fig. 3(f–g)). During the growth, the particles coalesce to form well-defined hierarchical structures (Fig. 3(h)), which with ~ 600 s of annealing, become somewhat disconnected, leaving nanogaps in between (Fig. 3(i)), similar to the 30 s annealed samples at 270 ºC (see Fig. 2). The images in Fig. 3(d–i) distinctly depict the dewetting course through particle nucleation and growth to form structures with varied characteristic length scales. The plot of the particle size estimated from these images shows a gradual increase with the annealing time, as found with the XRD data as well (Fig. 3(j–k) and S12). It may be noted that the crystallite sizes from XRD are somewhat smaller in value than the values from SEM, as the latter represents mostly the agglomerated structures. Based on the above observations, it becomes apparent that the dewetting process occurs through the drying and decomposition of the organic precursor leading to the nucleation of particles. The growth of nucleated particles and coalescence results in the dewetted structure. These processes follow each other with a significant overlap that closely follows the dewetting process of the self-assembly of nanoparticles in the colloidal dispersion [56, 57].

**Fig. 4:**
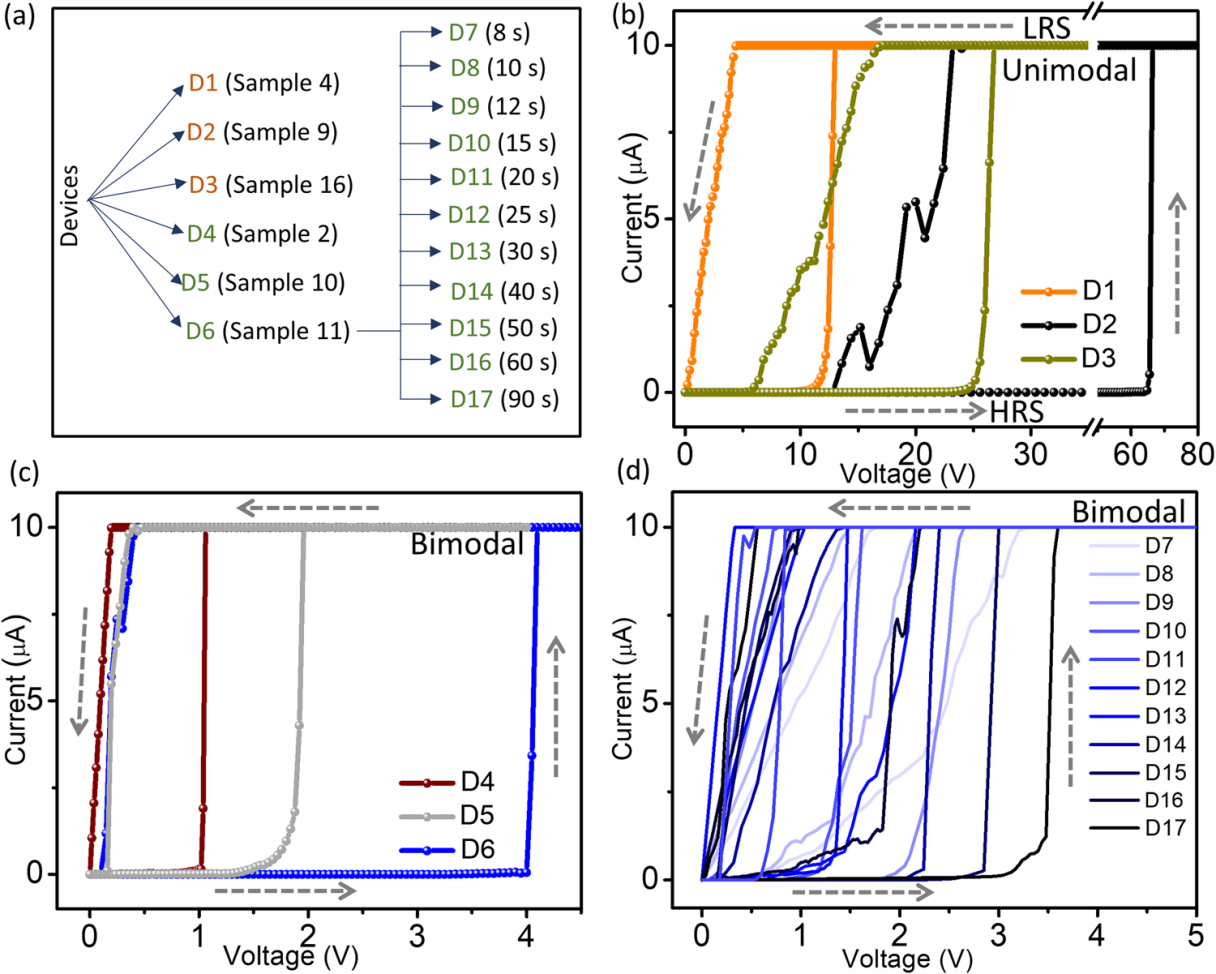
**a** Lists of samples with dewetted patterns used as active elements in devices (D). I-V characteristics of devices with **b** nearly unimodal and **c** bimodal distributions and **d** those obtained with different annealing times. Current compliance (I_CC_) is set at 10 µA in all cases

A few sets of optimized parameters were considered for the device fabrication as examples representing the different morphologies of the dewetted patterns. Accordingly, samples whose conditions were utilized to realize devices with the corresponding device and sample codes are shown in the lists (Fig. 4(a)), which will be followed throughout, otherwise mentioned. Further, devices were made by subsequently forming in-plane Au gap electrodes (see Fig. 1(a)), and an active area of ~ 600 × 40 µm^2^ was considered (as shown in the optical images in Fig. S13). The devices were consequently characterized by performing sequential I-V sweeps, specifically examining the threshold switching, the latter in the context of neuromorphic measurements. Figure 4(b–d) shows the resistive switching of the devices D1 to D17, exhibiting similar switching behaviors but with different threshold voltage (V_th_) values. Initially, the devices are in the high resistance state (HRS) due to disconnected Ag structures, and the conduction is dominated by tunneling. However, exceeding V_th_, the electric field might be sufficient to form conductive Ag filaments switching the device to the low resistance state (LRS) state [44]. Withdrawing the voltage causes filament breaking which could be due to the atomic surface diffusion driven by the minimization of the system energy [58]. The devices with nearly unimodal distributions in their active element (D1, D2, and D3-Fig. 4(b)) exhibit V_th_ of ~ 10, 70, and 25 V, respectively. However, the devices D4, D5, and D6 with bimodal distributions (Fig. 4(c)) exhibit V_th_ of ~ 1, 2, and 4 V, respectively. Thus, it is observed that devices with nearly unimodal distribution have higher V_th_ as compared to the bimodal distribution. This is true for devices obtained using different annealing times, especially D6, with V_th_ between 0.5 to 4 V (Fig. 4(d)). The devices show similar switching behavior in the negative applied voltages as well (Fig. S14).

The statistical analysis of the dewetted structures holds the clue for the observed variation in V_th_, as explained earlier. Comparing D1, D2, and D3, device D1 with V_th_ ~ 10 V hosts relatively low particle to particle distance and larger area particles (see Fig. 2(d) and S5(a)) compared to D2 (~ 70 V) and D3 (~ 25 V). It is also noteworthy that the recovery voltage for devices D2 and D3 is higher compared to that of D1, which may be attributed to the formation of weaker filaments connecting relatively smaller particles. Now, when the devices D4 to D6 are compared, D6 (~ 4 V) has a larger island to island distance, smaller island, and particle areas, thus having a higher V_th_ than D4 (~ 1 V) and D5 (~ 2 V). The above observations denote that a smaller distance between the particles (islands) and a larger area of the particles (islands) result in a lower V_th_. Also, with annealing time, the V_th_ increases due to the increased distance between the particles and islands (Fig. S15).

**Fig. 5:**
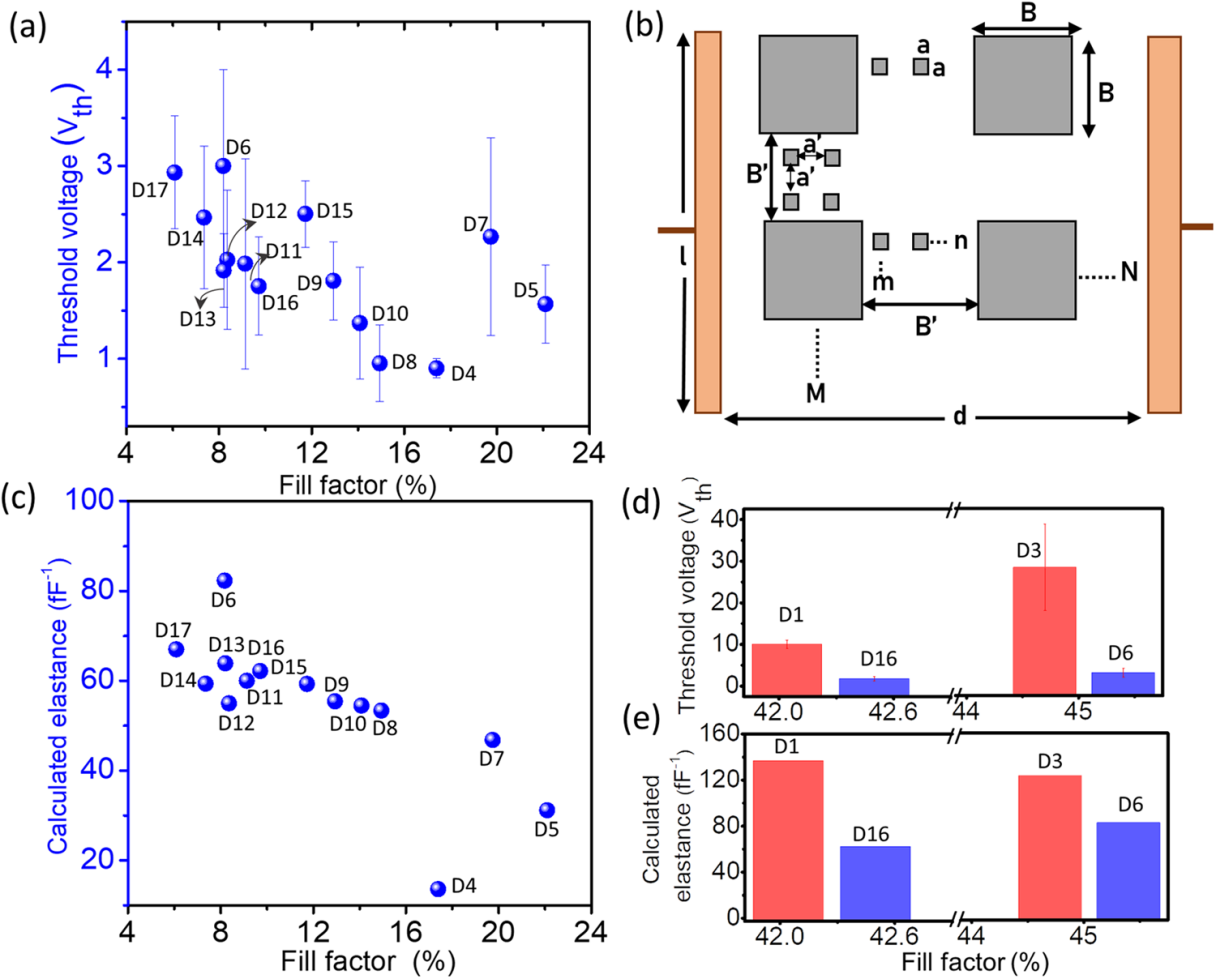
**a** V_th_ values versus the Ag fill factors in devices D4 to D17. The error bars indicate the deviation of the V_th_ for three devices in each case. **b** Schematic of a device-equivalent, parallel plate capacitor model with gray squares indicating the presence of Ag islands (large with $B$ sides) and nanoparticles (small with $a$ sides). The distance between the particles and the Ag islands are ${a}{\prime}$ and ${B}{\prime}$, respectively. **c** Calculated elastance versus the Ag fill factor for devices D4 to D17. **d** Variation in V_th_ for devices with similar FF of ~ 42% for D1 and D16 and ~ 45% for D3 and D6. **e** Variation in the elastance values for devices with similar FF. However, D1 and D3 are nearly unimodal (red bars), and D16 and D6 (blue) are bimodal

Specifically, to further compare the V_th_ of the different devices (D4 to D17), the areal (2D) Ag fill factor (FF) was estimated using ImageJ as a fractional sum of the FF of Ag islands and that of nanoparticles (method of calculation is given in the). It was observed that the FF of Ag islands significantly affected the V_th_ (Fig. S16). Thus, with the increase in the FF of islands, the V_th_ decreased (Fig. 5(a)). Also, a capacitance model was developed to relate the V_th_ with the microscopic aspects considering the device to be a parallel plate capacitor with dielectric consisting of air and isolated metal particles (Fig. 5(b)). The distance between the plates (in this case, the Au electrodes) is $d$, the length of the electrodes is $l$, and the thickness is $t$ (not shown). The air gap is filled with Ag nanostructures (islands and nanoparticles) which, for simplicity, are shown as small and big squares of sides $a$ and $B$, respectively, with interparticle distances of ${a}{\prime}$ and ${B}{\prime}$, respectively (all assumed to be constant), such $M$ and $N$ big units (parallel and perpendicular respectively to the electrode shown), and m and n small units repeating themselves; the resultant capacitance will be,

1 $$C= {C}_{0}\left(1+ \frac{{a}^{2}}{{a}{\prime}(a+{a}{\prime})}\right)\left(1+ \frac{{B}^{2}}{{B}{\prime}(B+{B}{\prime})}\right)$$

where ${C}_{0}$ is the capacitance of the device with no Ag nanostructures (only air gap). Based on this equation, $C$ values were calculated using the morphological parameters derived from microscopy images of the dewetted patterns present in devices (ImageJ was used to obtain the parameters $a$, $B$, ${a}{\prime}$, and ${B}{\prime}$). For convenience, the inverse of $C$, called elastance $S$ is considered since $S$ is directly related to the V_th_ (details are given in the). The variation of $a$, $B$, ${a}{\prime}$, and ${B}{\prime}$ with V_th_ is shown in Fig. S17.

Interestingly, the calculated S values decreased with an increase in FF (Fig. 5(c)), similar to V_th_. In addition, though D1 and D16, D3 and D6 had similar overall FF of ~ 42 and 45%, respectively, D1 and D3 being nearly unimodal, exhibited higher V_th_ than that of bimodal D16 and D6 (Fig. 5(d–e)). Excitingly, the elastance values calculated from the model were also higher for D1 and D3 and lower for D16 and D6, respectively. It is worth noting that the device D2, which had the highest V_th_ (Fig. 4(b)), also had the highest elastance value (~ 147 fF^−1^). Furthermore, it is appreciable that the elastance values obtained from the model are in accordance with the experimental V_th_ values. In the future, such a model can be considered as a basic tool for identifying suitable device parameters to obtain better neuromorphic functionalities.

**Fig. 6:**
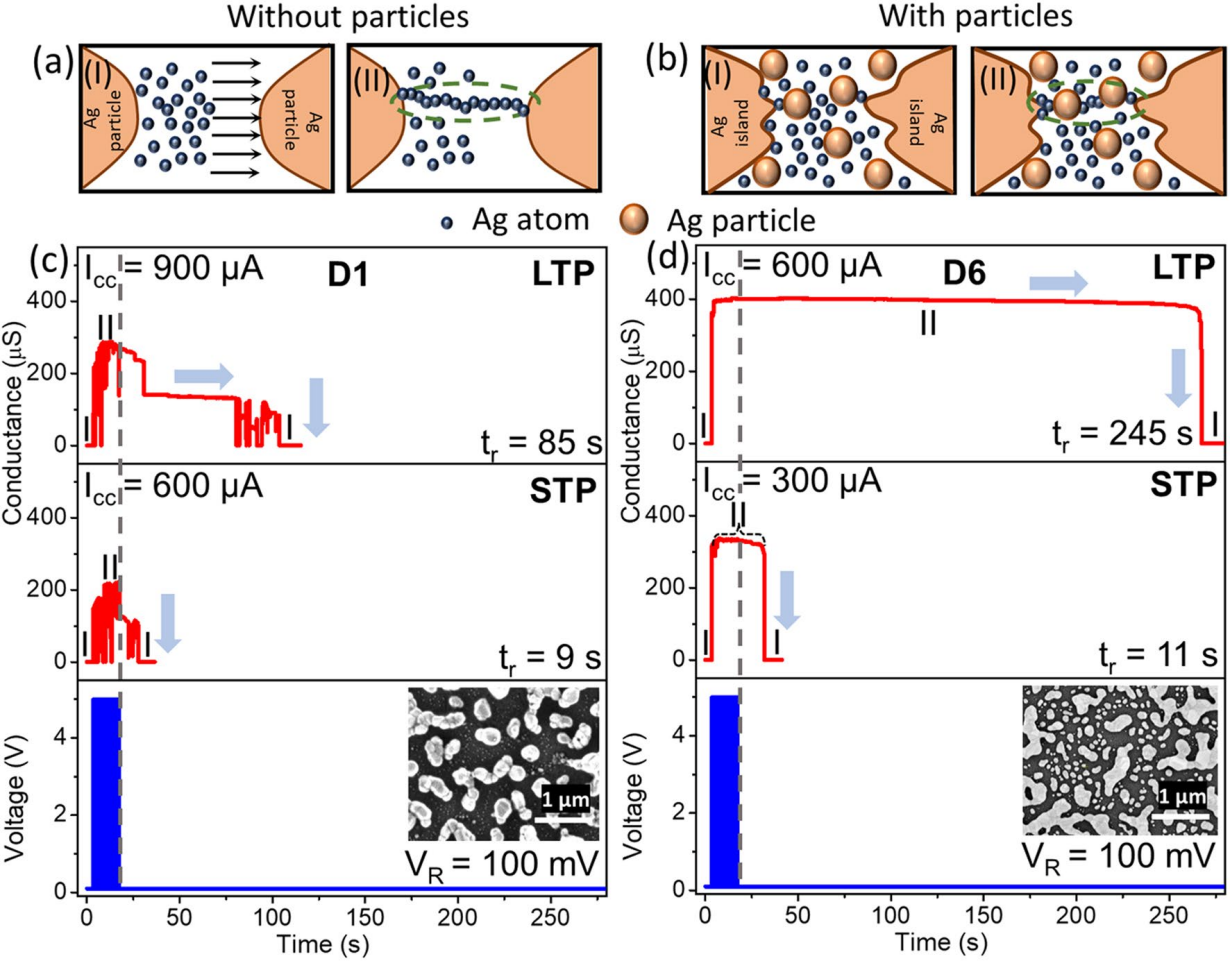
**a, b** (states I and II) Schematic showing the state of the nearly unimodal and the bimodal device before and after pulsing, respectively. The green dotted circle represents the conductive filamentary path possibly formed during pulsing (state II). Short-term potentiation (STP) and long-term potentiation (LTP) were emulated in the devices **c** D1 at the set I_CC_ of 600 and 900 µA while exhibiting a retention time (t_r_) of 9 and 85 s, respectively, **d** D6 at the set I_CC_ of 300 and 600 µA while exhibiting a t_r_ of 11 and 245 s, respectively. The bottom panel in c and d represents 50 pulses of 5 V with 200 ms width as well as the interval. The reading voltage is 100 mV

To further emulate and compare the neuromorphic behavior of the devices, a series of voltage pulses are applied to the devices. Figures 6(a) and (b) show schematic of the devices without and with particles, respectively, indicating the state of the devices before (state I), during, and after (state II) pulsing. Stage I, which represents the HRS of the devices, switches to Stage II (LRS) due to the application of voltage pulses leading to conductive filamentary path formations. As the voltage pulses are withdrawn, based on the stability and strength of the filaments, the filaments break and relax back to state I (with the possibility of a slight deviation).

The behavior of the device for the voltage pulses was exploited to emulate STP and LTP. Such behavior in the biological systems is attributed to the strength and stability of the synapses [59], and in the device, it can be attributed to the strength and stability of the filaments. To understand the STP and LTP behavior shown by devices exhibiting different hierarchical structures, representative bimodal (D6- with particles) and nearly unimodal (D1- without particles) devices were considered. At a background reading voltage of 100 mV (V_R_), the devices were fed with 50 pulses of amplitude 5 V with 200 ms as both width and interval, at different I_CC_ (current compliance), as shown in the bottom panel of Figs. 6(c) and (d) (the zoomed-in image of the voltage pulses is shown in Fig. S18). The set I_CC_ decides the strength and stability of the filaments formed, and the behavior of the devices after pulsing was observed with a V_R_ of 100 mV. Figure 6(c) shows the STP and LTP behavior of D1, where the conductance of the device reaches a certain state during the application of voltage pulses and, after the cessation of the voltage pulses, remains in the LRS state for a few seconds (~ 9 s) in the case of STP and for more than a minute (~ 85 s) in the case of LTP. The conductance values were ~ 200 and 300 µS for STP and LTP, respectively. On the contrary, device D6 shows an STP of ~ 11 s and an LTP of ~ 245 s with the conductance values reaching ~ 300 and 400 µS, respectively (Fig. 6(d)). From there, it can be clearly observed that the bimodal device D6 shows better retention and higher conductance values and requires lower I_CC_ to achieve the STP and LTP behaviors. Also, it can be observed that even with utilizing higher I_CC_, the nearly unimodal device D1 showed lower retention and conductance values. Indeed, in D1, after the pulse removal, the conductance reached intermediate states both in STP and LTP. With all these observations, it appears that due to the lack of hierarchy and farther particles in D1, the strength and stability of the filaments are weaker as opposed to that in D6 which exhibits a commendable hierarchy, and this is a possible reason for the observed differences in STP and LTP behaviors of D1 and D6.

**Fig. 7:**
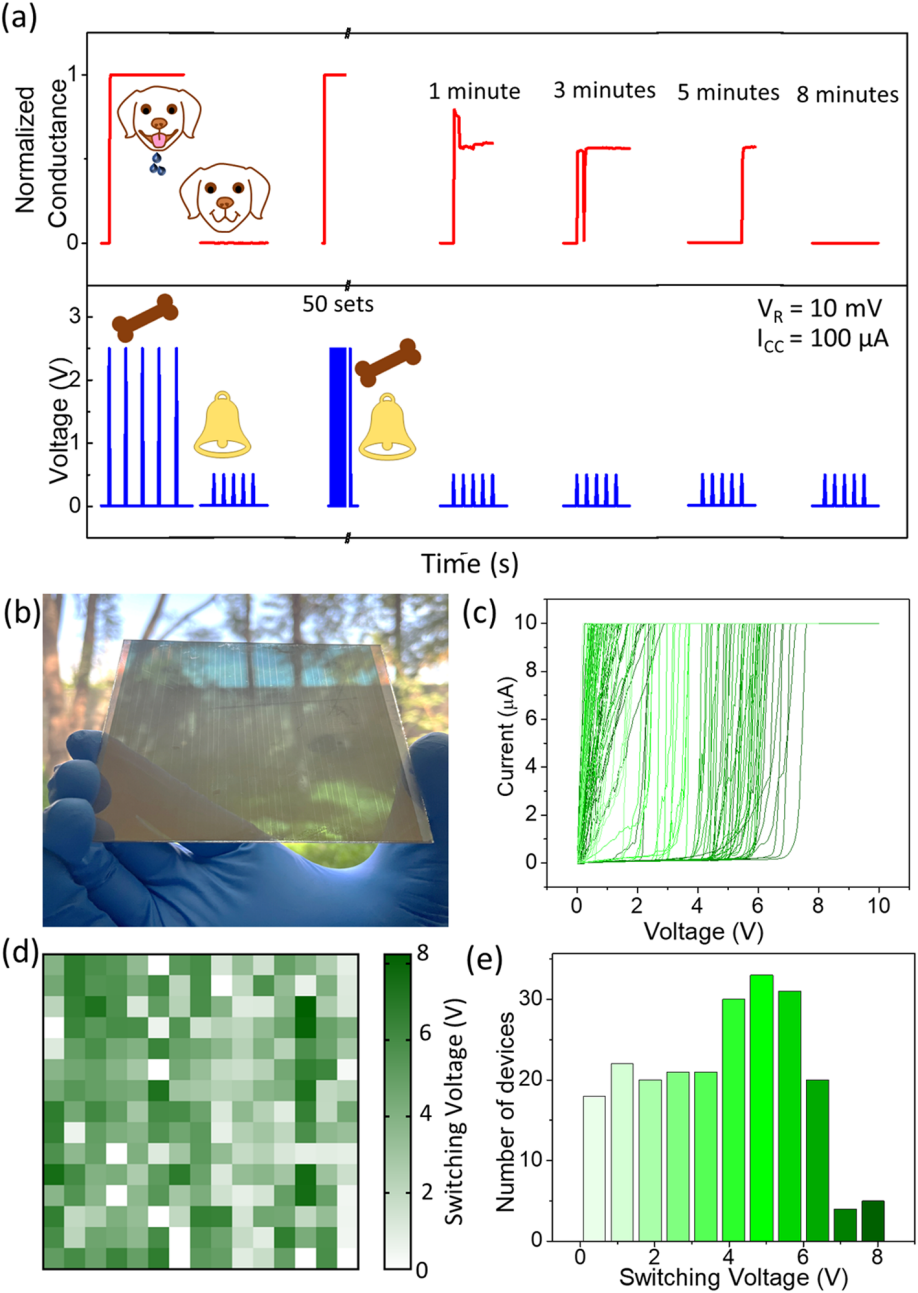
**a** Associative learning emulated in the device, D6. Food (5 pulses, 2.5 V) and bell (5 pulses, 0.5 V) signals were fed to the device consecutively. The device responded to the food signal but not to the bell signal. During training, the food and bell (50 sets) signals were fed with no gap. After training, the device responds to the bell signal alone with a retention of up to 8 min. All the pulses were of 1 s width and interval. **b** Photograph of the large area (~ 10 × 10 cm^2^) dewetted Ag device containing 225 devices each of ~ 600 × 40 µm^2^ area. **c** I–V characteristics of 75 devices; only 75 out of 225 curves are shown for the sake of clarity. **d** Color map of switching voltages of the 225 devices. **e** Histogram showing the variation in the switching voltage for the devices

We then embarked on emulating one of the complex cognitive processes namely the associative learning, following the Pavlov’s dog experiment [60], which has become a benchmark among neuromorphic related studies [24, 34–37]. In Pavlov’s dog experiment, the dog salivates at the sight of food, but it does not respond to a ringing bell that is of no interest and is therefore an unconditioned stimulus. However, when the dog is exposed to both the food and the ringing bell consecutively for a few times, the dog responds by salivating only to the bell, even in the absence of food. This is because the dog learns to associate the unconditioned stimulus (the ringing bell) with the presence of food (conditioned stimulus). The same was emulated using the D6 device (Fig. 7(a)), where five pulses of 2.5 and 0.5 V were assigned to food and bell signals, respectively. Initially, the device responded to the food pulse (increased conductance) but did not respond to the bell pulse (no conductance change) as expected. Later, 50 sets of training pulses were applied to the device, with each set containing five pulses of food and bell consecutively. After the training, the device responded to the bell signal alone, indicating a successful association of the bell and the food signal that lasted for ~ 8 min. The ability of the device to exhibit associative learning is due to the diversity of filaments in the diverse nanogaps. The food pulse bridges smaller nanogaps that can handle the set I_CC_ (100 µA). However, the conical filaments bridging the larger nanogaps with low ampacity and thin dimensions also get stabilized during training due to the presence of the bell pulses. This results in the response of the device for bell pulse alone post-training [44].

Scalability, an important aspect of integration, has been demonstrated by fabricating the device on an area of ~ 10 × 10 cm^2^ with the same dewetting conditions as D4 (see the photograph in Fig. 7(b)). Fig. S19 contains the SEM image of the dewetted pattern in the large device. Figure 7(c) shows the switching voltages ranging from 2 to 7 V for 75 devices of area ~ 40 × 600 µm^2^ on the ~ 10 × 10 cm^2^ substrate. The color map shows the aerial view of the spread of switching voltages across devices (Fig. 7(d)). The histogram of the 225 devices indicates that most of the devices switch below 7 V (Fig. 7(e)), which is remarkable. It is noteworthy that the device is easily scalable with single-step processing and without the involvement of lithography. Interestingly, 96% of the devices show the switching behavior which is quite promising.

**Fig. 8:**
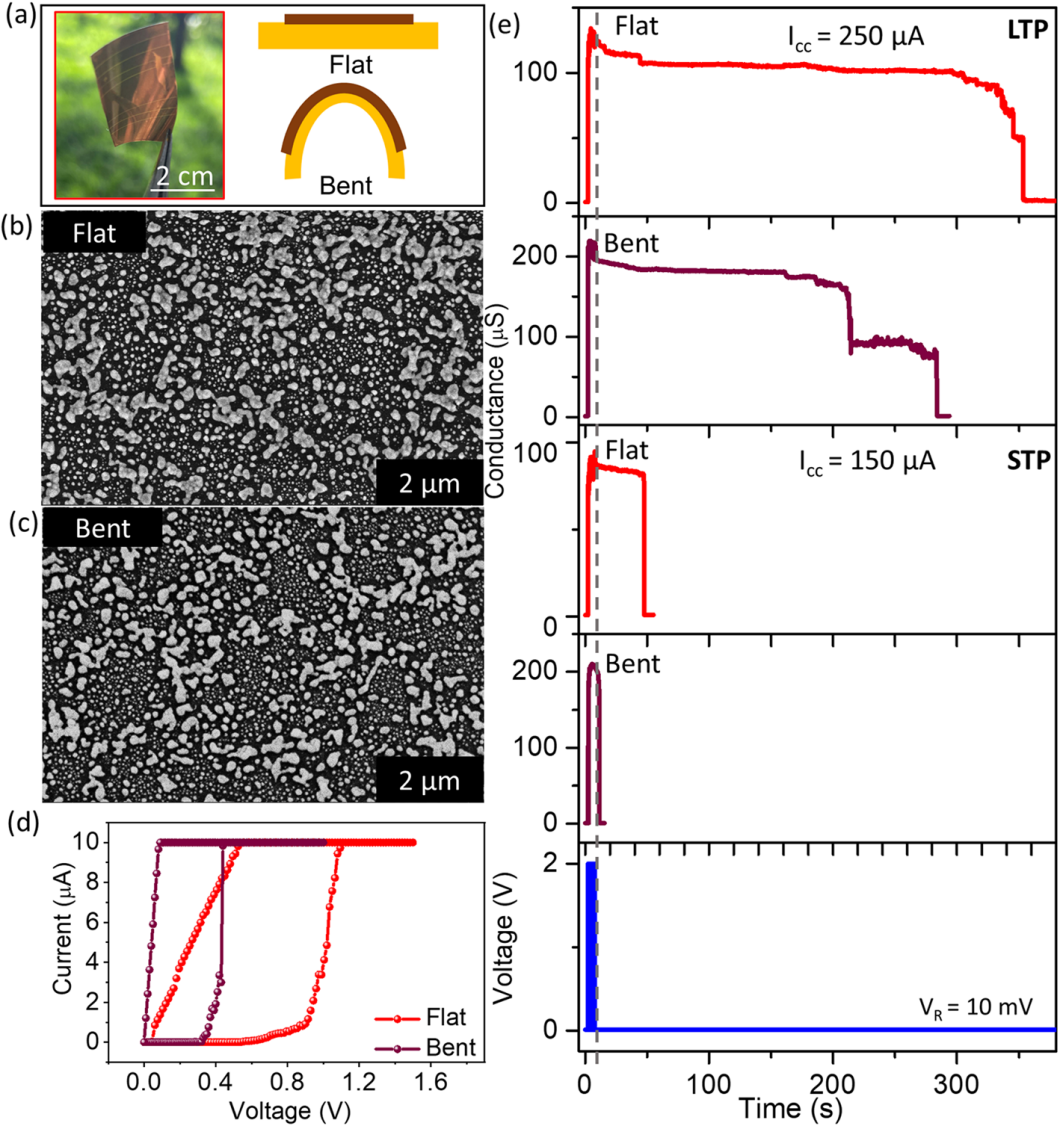
**a** Photograph of the flexible device along with the schematic of the device in the flat and bent states. **b–c** SEM image and **d** I-V characteristics of the device in the flat and bent states. **e** STP and LTP emulated in the flexible device (20 pulses, 2 V: bottom panel) at I_CC_ of 150 µA (STP) and 250 µA (LTP), in the flat and bent states

Flexible electronics, gaining significant advancements in the recent past demand the fabrication of flexible neuromorphic devices. Here given the simplicity of the chemical synthetic process, a flexible device was also fabricated on a Kapton substrate (Fig. 8(a)) by dewetting and depositing the Au electrodes, as explained in Fig. 1(a). The conditions for dewetting are the same as that of D4, with the annealing time being 25 s. The SEM images of the device in the flat and bent states (Fig. 8(b–c)) show the presence of disconnected particles. Further, the I-V characteristics of the device in the flat and bent states show the device switching at ~ 0.8 and 0.4 V, respectively (Fig. 8(d)). This confirmed the working of the device both in the flat and bent states. The devices further emulated the STP and LTP behavior. A pulsing sequence of 20 pulses with 2 V amplitude and 200 ms pulse width and interval was used at 150 and 250 µA I_CC_ for emulating the STP and LTP behaviors, respectively, in both the flat and bent states (Fig. 8(e)). The flat and bent states at STP showed a retention of ~ 30 and 5 s, respectively, while at LTP showed a retention of ~ 345 and 275 s, respectively. This shows the potential of the device to work as a flexible device as well.

## Conclusions

We have reported a self-formed Ag device fabricated by a chemical route as an active element in neuromorphic devices emulating the synaptic functionalities exhibited by the biological systems. The dewetting parameters like the spin coating speed (1000 to 6000 rpm), precursor:solvent (0 to 0.4), and IPA:water (0 to 1) ratios were optimized to obtain hierarchical structures which seemed important, given the possible similarity to those observed in the neural system. The hierarchical structures with bimodal distribution were obtained with lower spin coating speed (< 3000 rpm), higher IPA:water ratio (> 0.8), and higher precursor to solvent ratio (> 0.3). For comparison, selected bimodal and nearly unimodal dewetted patterns were analyzed statistically, and an insight into the dewetting mechanism was provided by a microscopic study. Further, various devices with bimodal distributions were electrically characterized by finding the threshold voltage for resistive switching, V_th_. For understanding the variation in V_th_ in relation to the dewetted structures, the fill factors (fraction of the active area filled with Ag particles and islands) were calculated. Interestingly, V_th_ increased with the decrease in the fill factor. Also, a capacitance model was developed to consider all the microscopic parameters, such as the distance between the particles and islands, the size of the particles and islands affecting the V_th_. Interestingly, the elastance values increased, showing a similar trend to V_th_ with a decrease in fill factor as expected. After analyzing the dewetted structures, the STP and LTP synaptic functionalities were emulated in one representative bimodal and nearly unimodal device. The STP and the LTP behaviors were found to be superior in the bimodal device stressing the importance of hierarchical structures. One of the cognitive behaviors, associative learning, was also emulated in the bimodal device. Scalability, a crucial aspect for integration, is demonstrated by fabricating more than 1000 devices on a ~ 10 × 10 cm^2^ substrate using the established chemical recipe. Among the 225 devices tested, 96% showed the switching behavior. In addition, a device was also fabricated on a flexible substrate, and the synaptic functionalities (STP and LTP) were demonstrated. The emulation of synaptic functionalities (STP and LTP), cognitive behavior, along with the crucial integration aspects such as scalability and flexibility of devices fabricated by a simple chemical route, is promising for their utilization in neuromorphic circuitry.

### Supplementary Information

**Additional file 1:** Supplementary Information (SI).

**Additional file 2:** Dewetting video S1.

## Data Availability

All data generated or analyzed during this study are included in this article and its supplementary information file.
