# Supplementary material for: A scalable solution recipe for a Ag-based neuromorphic device
Source: Discov Nano. 2023 Oct 9;18(1):124. doi: 10.1186/s11671-023-03906-5 (PMC10562349; doi:10.1186/s11671-023-03906-5)
Supplement: Supplementary file 1 — Additional file 1: Supplementary Information (SI). [file 11671_2023_3906_MOESM1_ESM.docx]

**Supplementary Information**

**A scalable solution recipe for a Ag-based neuromorphic device**

Tejaswini S. Rao­, Indrajit Mondal, Bharath Bannur, Giridhar U. Kulkarni*

Chemistry & Physics of Materials Unit, Jawaharlal Nehru Centre for Advanced Scientific Research, Jakkur P.O., Bangalore - 560064, India

***** Email: [kulkarni@jncasr.ac.in](mailto:kulkarni@jncasr.ac.in)


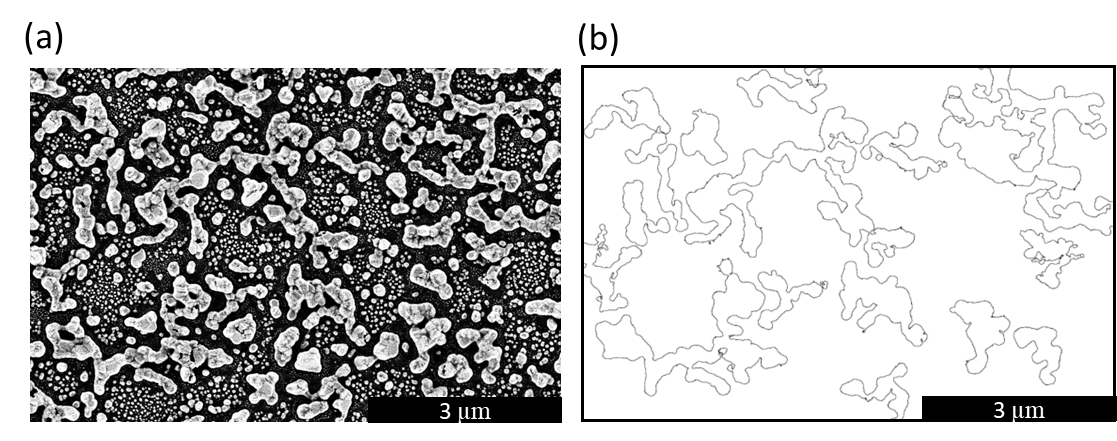


**Fig. S1** **(a)** SEM image of the dewetted Ag film. **(b)** Particles of area > 0.3 µm^2^ are considered to be islands as indicated and others as particles.


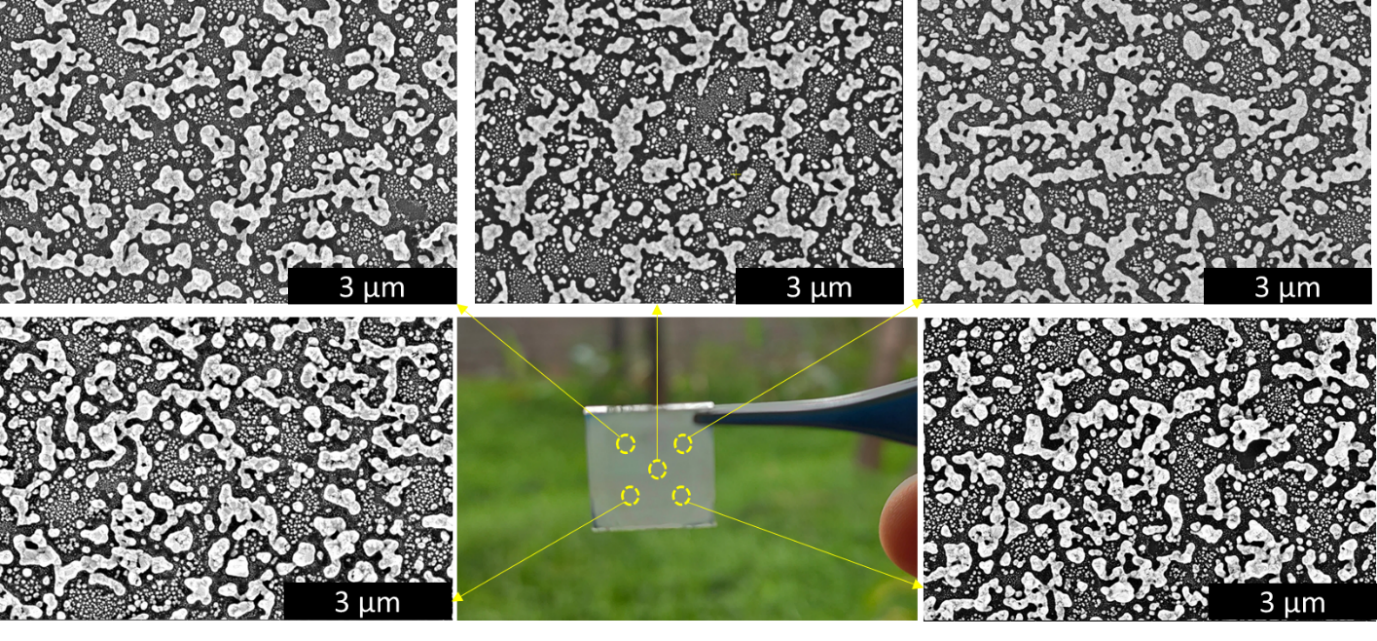


**Fig. S2** SEM images on different regions of a 1×1 cm^2^ substrate showing uniformity of the dewetting pattern.


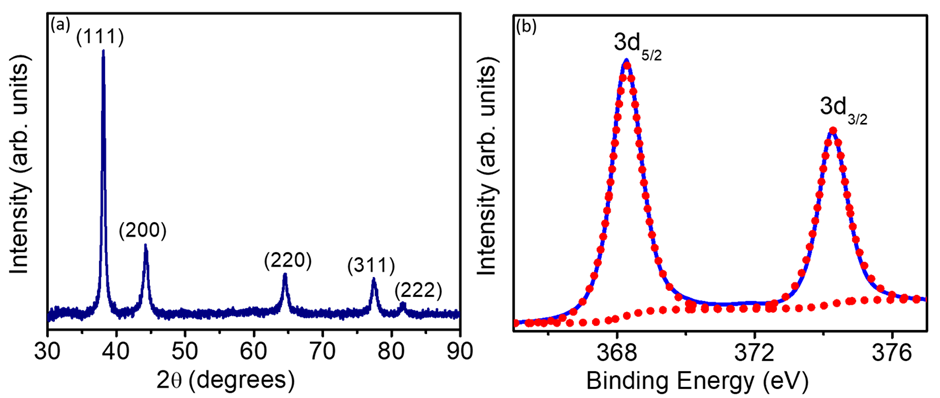


**Fig. S3** **(a)** XRD pattern of the film showing prominent (111) peaks. The peaks match with the face-centered cubic (fcc) structure of silver (JCPDS:04-0783) **(b)** XPS analysis showing the peaks for core level Ag 3d.

| Sample number | Solvent* | Ag precursor: solvent ratio** | Spin coating speed (RPM) | SEM image | Observations | Type of hierarchy | Device code |
| --- | --- | --- | --- | --- | --- | --- | --- |
| 1 | - | 1:0 | 1000 | ✔ | Conducting even after 2 hours | - |  |
| 2 | IPA |  | 1000 | ✔ | Continuous dewetted film | Bimodal | D4 |
| 3 | IPA |  | 2000 | ✔ | Continuous dewetted film | Bimodal |  |
| 4 | IPA | 1:2 | 3000 | ✔ | Continuous dewetted film | Nearly Unimodal | D1 |
| 5 | IPA |  | 4000 | ✔ | Continuous dewetted film | Nearly Unimodal |  |
| 6 | IPA |  | 5000 | ✔ | Continuous dewetted film | Nearly Unimodal |  |
| 7 | IPA |  | 6000 | ✔ | Continuous dewetted film | Nearly Unimodal |  |
| 8 | IPA | 1:4 | 1000 | ✔ | Continuous dewetted film | Nearly Unimodal |  |
| 9 | IPA | 1:6 | 1000 | ✔ | Continuous dewetted film | Nearly Unimodal | D2 |
| 10 | 95:5=IPA:water |  |  | ✔ | Continuous dewetted film | Bimodal | D5 |
| 11 | 85:15=IPA:water |  |  | ✔ | Continuous dewetted film | Bimodal | D6 |
| 12 | 60:40=IPA:water |  |  | ✖ | Wettability was poor and continuous film was not obtained | - |  |
| 13 | 40:60=IPA:water | 1:2 | 1000 | ✖ | Wettability was poor and continuous film was not obtained | - |  |
| 14 | 20:80=IPA:water |  |  | ✖ | Wettability was poor and continuous film was not obtained | - |  |
| 15 | water |  |  | ✖ | Precursor solution itself turned turbid | - |  |
| 16 | 85:15=IPA:water | 1:4 |  | ✔ | Continuous dewetted film | Nearly Unimodal | D3 |
| 17 | 85:15=IPA:water | 1:6 | 1000 | ✔ | Continuous dewetted film | Nearly Unimodal |  |
| 18 | 85:15=IPA:water | 1:8 |  | ✔ | Continuous dewetted film | Nearly Unimodal |  |

**Table S1 Optimization of dewetting parameters**

*Solvent is taken as IPA:water ratio in Figure 1b. Only IPA is considered to be 1 and with dilution the ratio varies as 0.95, 0.85, 0.6 etc.

**Ag precursor to solvent ratio is taken as precursor:solvent ratio in Figure 1b. For example: if the ratio is 1:2, then the precursor ratio is 1/3 = 0.33 and so on.


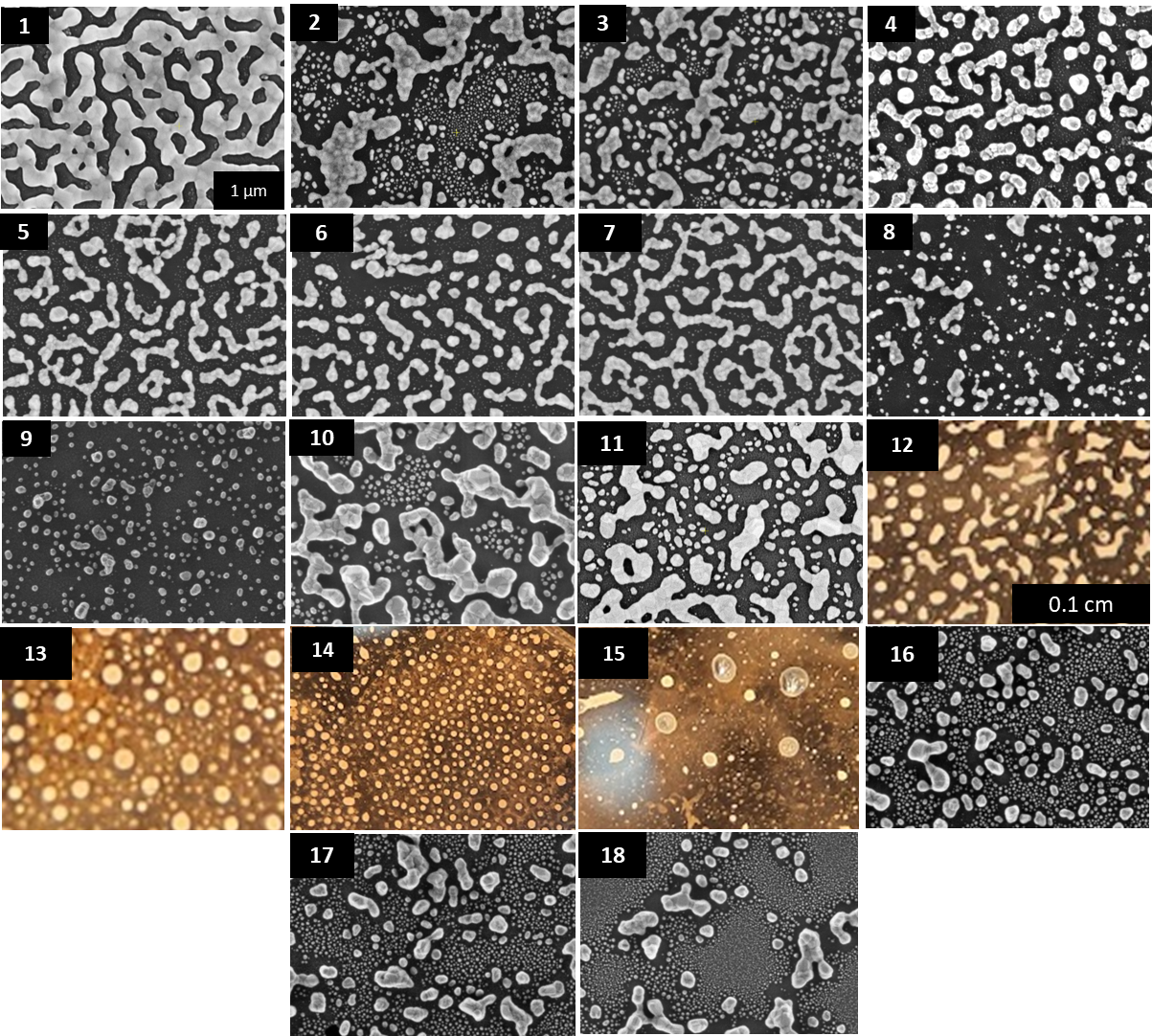


**Fig. S4** SEM and optical images of samples 1 to 18 prepared by using the parameters mentioned in Table S1. Scale bar: 1 µm and 0.1 cm applies to all the SEM and optical images, respectively.


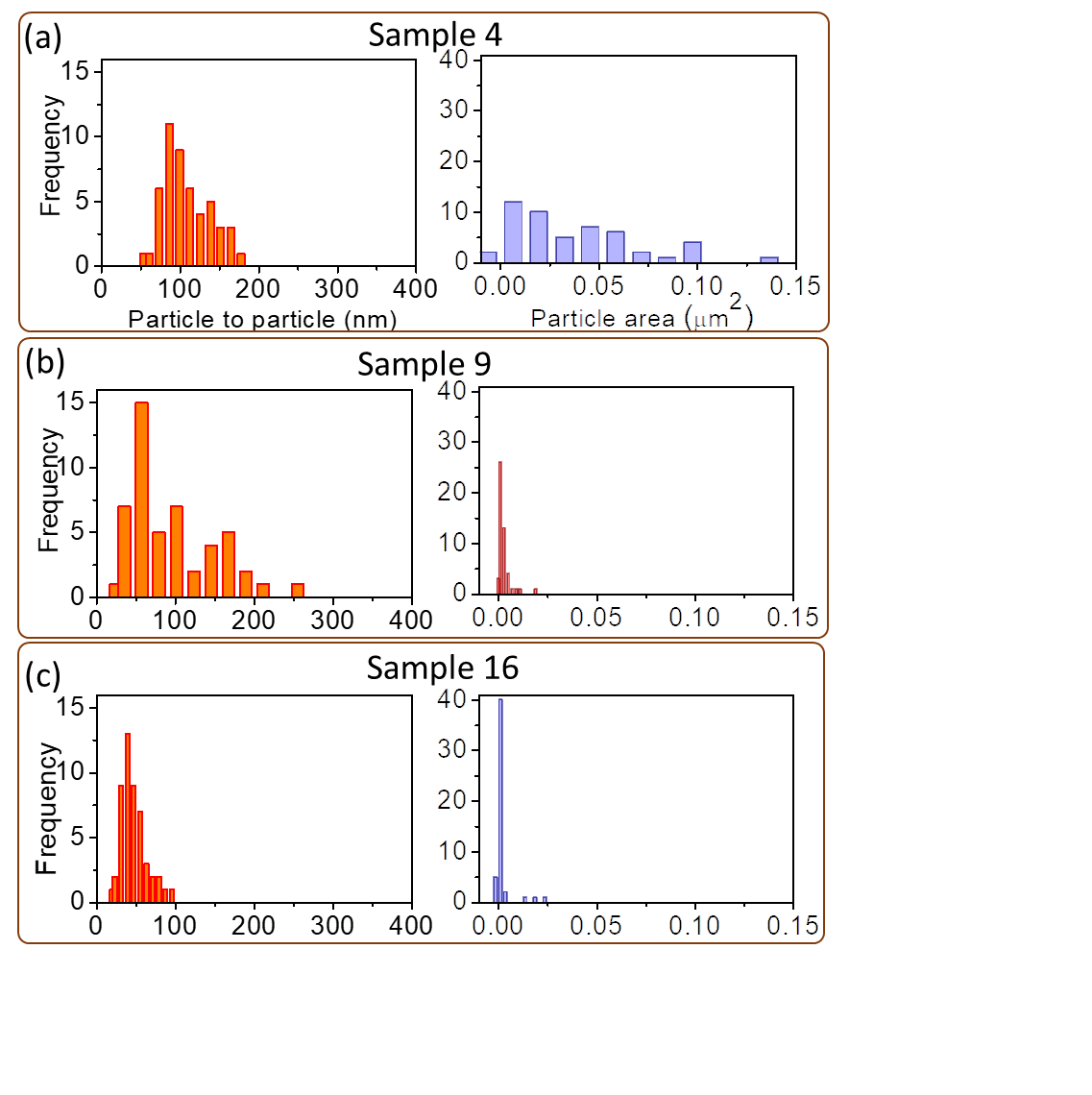


**Fig. S5** **(a-c)** Histograms corresponding to particle to particle distance, and particle area of the dewetted patterns of samples 4, 9, and 16 are shown in Figure 2d-f.


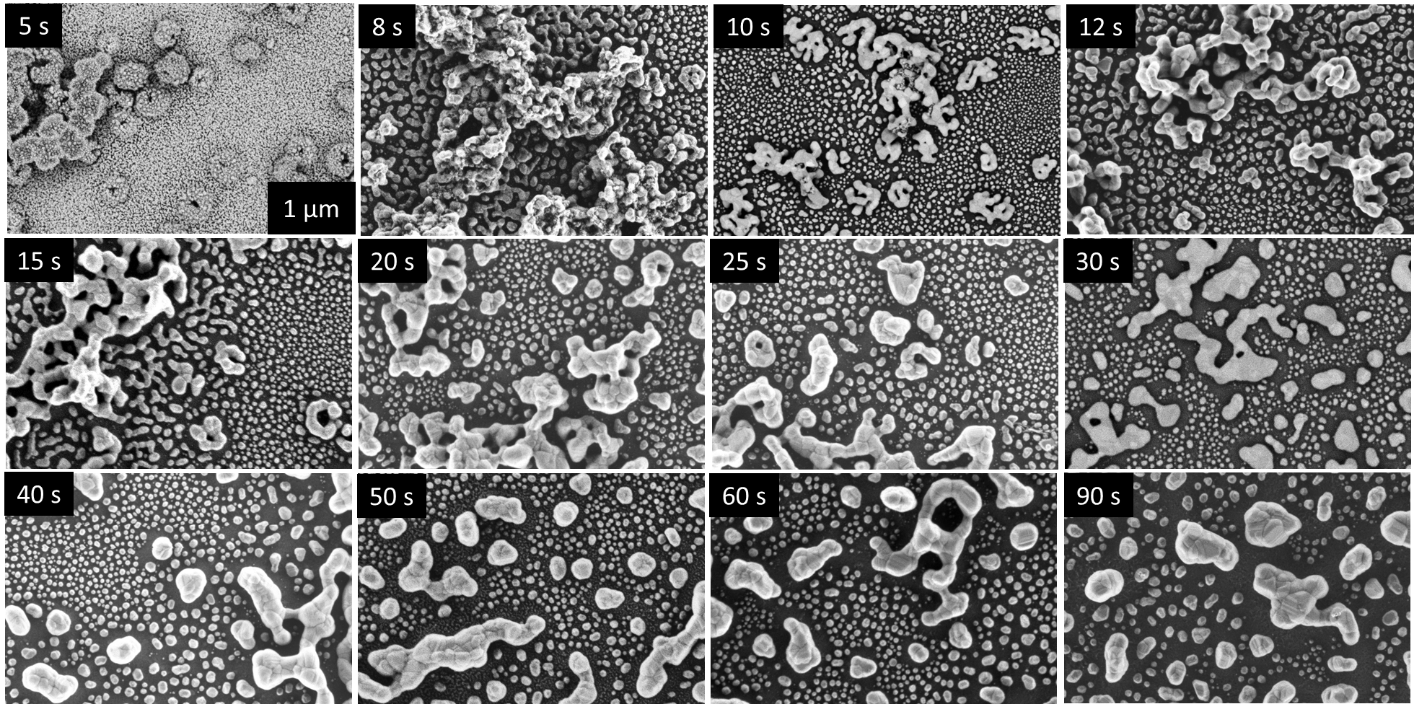


**Fig. S6** SEM images of samples with different annealing times. The scale bar applies to all the images.


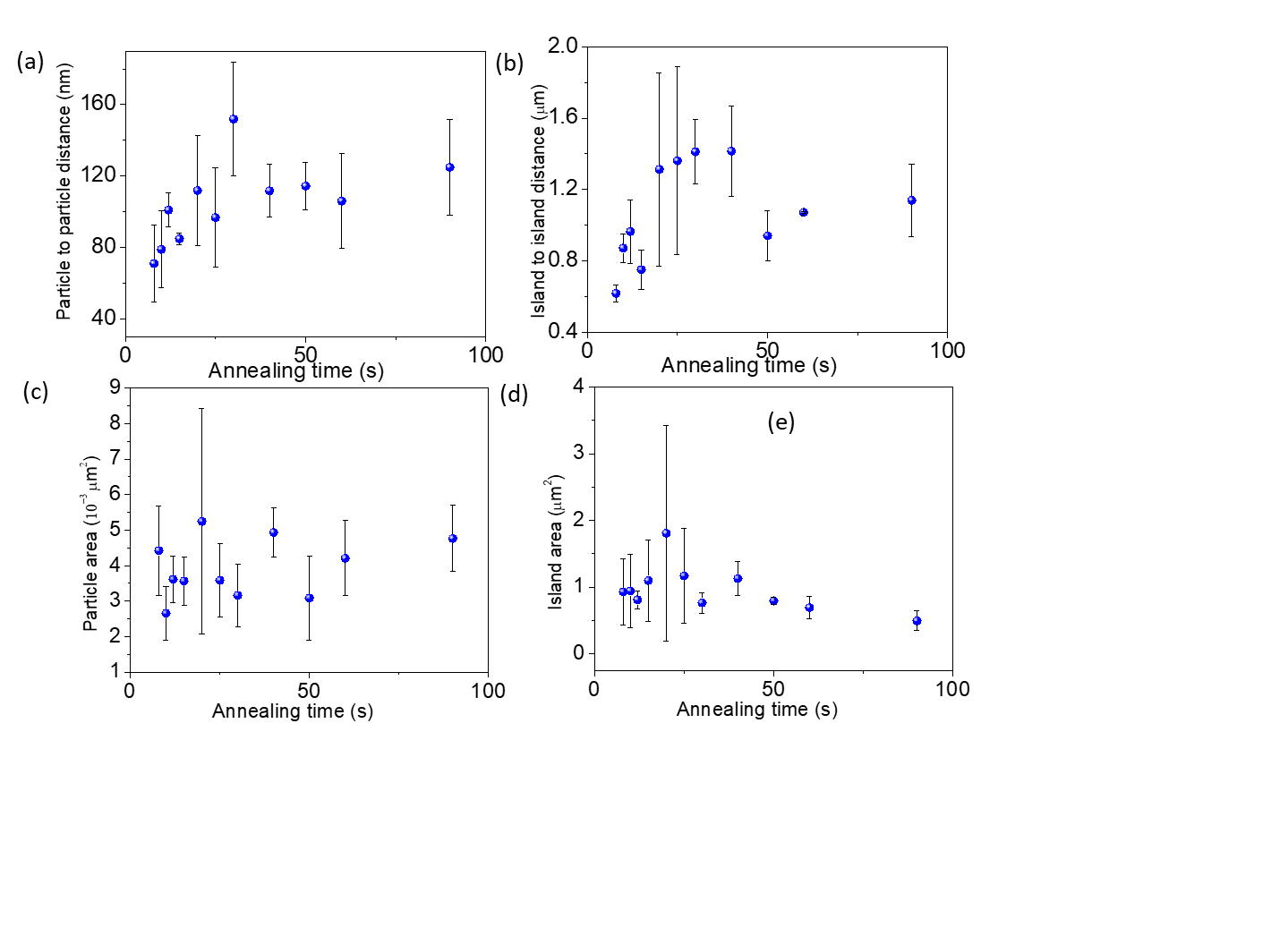
**Fig. S7** The particles of the area above > 0.3 µm^2^ are considered as islands and others as particles for simplicity. Variation of the **(a)** particle to particle distance **(b)** island to island distance **(c)** particle area **(d)** island area for different annealing times.


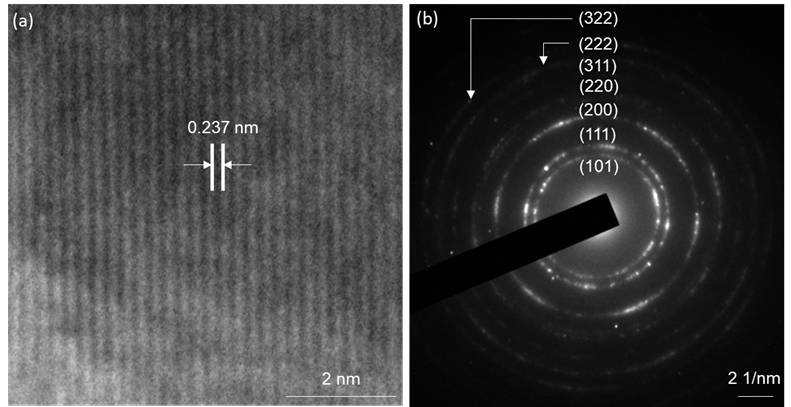


**Fig. S8** **(a)** HRTEM image and **(b)** SAED pattern of the Ag particle from the 600 s annealed Ag film. The lattice fringes in (a) corresponds to Ag (111) peak.


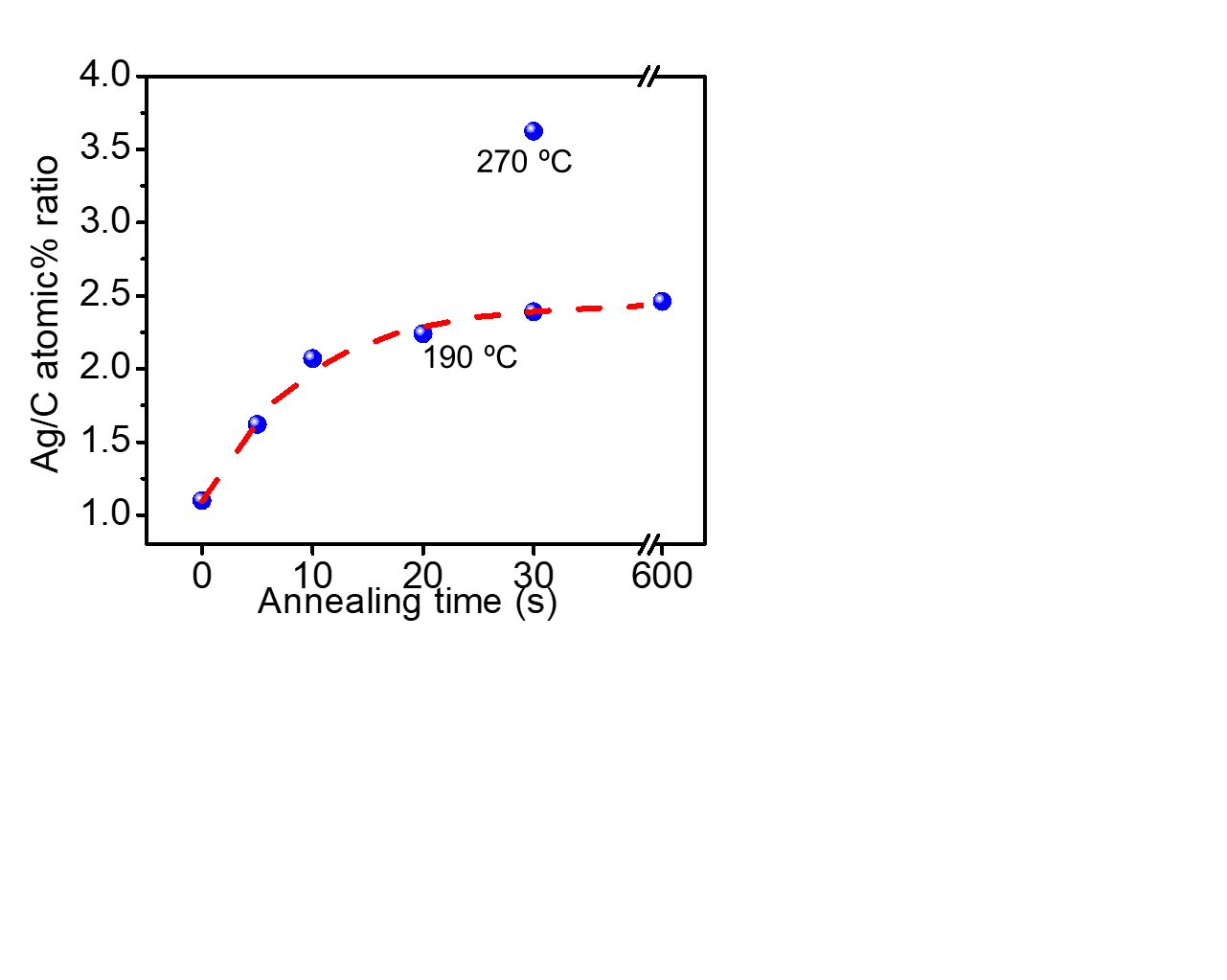


**Fig. S9** Variation in Ag/C atomic percentage showing the decrease in carbon content or in organic species with annealing time.

***In situ* optical microscopy studies**

To facilitate the *in-situ* study, an optically transparent fluorine doped tin oxide (FTO) film was used as a heater whose heating profile with the applied voltage is shown in Fig. S10(a). The infrared (IR) thermal image of a glass substrate placed on the heater shows that the glass obtains an average temperature of ~190 °C for an applied voltage of 10 V (Fig. S10(b)). The variation in the film appearance and morphology with time is shown in the video S1 and Figs. S11(a-d).


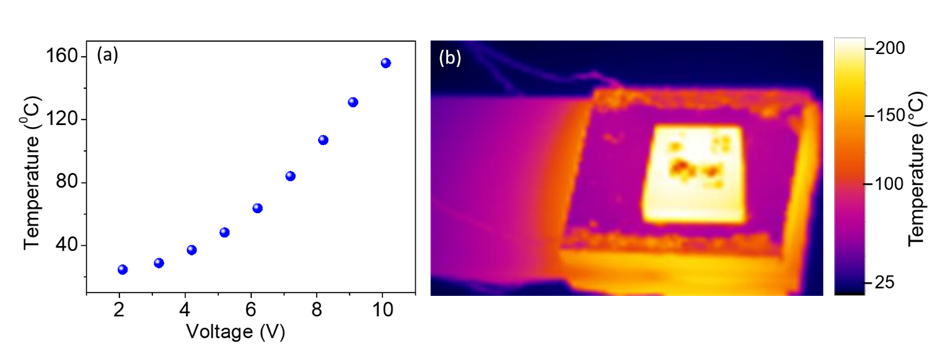


**Fig. S10** **(a)** Heating profile of FTO heater with increasing voltage. **(b)** Thermal image of a glass substrate placed on the FTO heater for an applied voltage of 10 V.


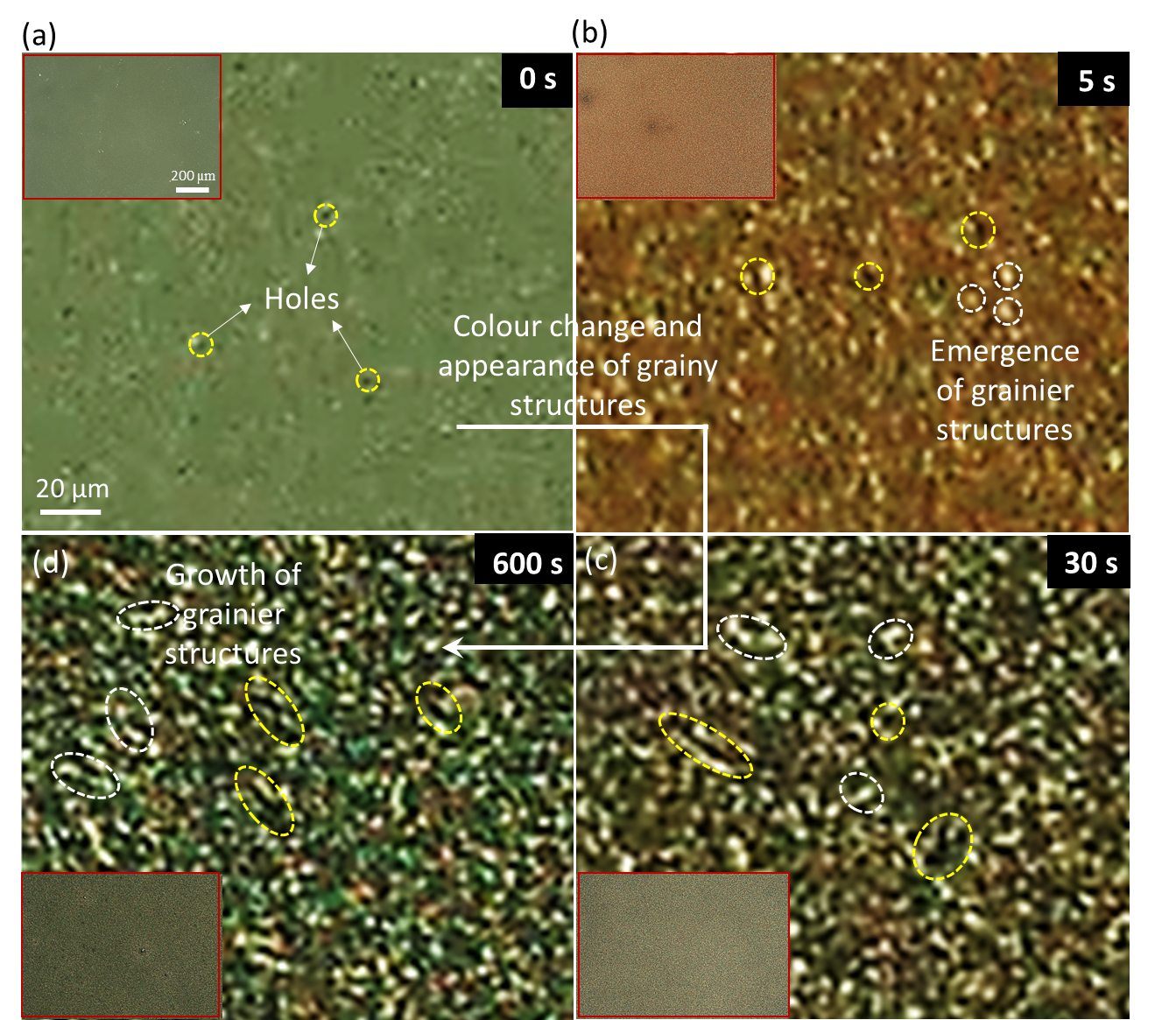


**Fig. S11** **(a)-(d)** Optical microscopy images captured at different annealing times during the in-situ study showing the color changes and the evolution of particles with annealing, (Scale bar: 20 µm). Inset shows the images in lower magnification.

The process of dewetting can start at any time after the precursor solution is spread on the substrate which is the reason for the appearance of holes (yellow dotted circles) just after the spin coating process (t = 0 s). However, the holes grow as the film is annealed at 190 ºC which is observed at the annealing time, t = 5 s, and small grainier structures (white dotted circles) begin to appear. Also, the color of the film is changing from green to brown which can be an indication of decomposition of the organic constituents. At t = 30 s, the holes and the grainier structures grow bigger and further growth of grainier structures can be seen at t = 600 s. The color of the film is almost similar to that at t = 0 s which might be due to the loss of organic constituents, leading to metallization.


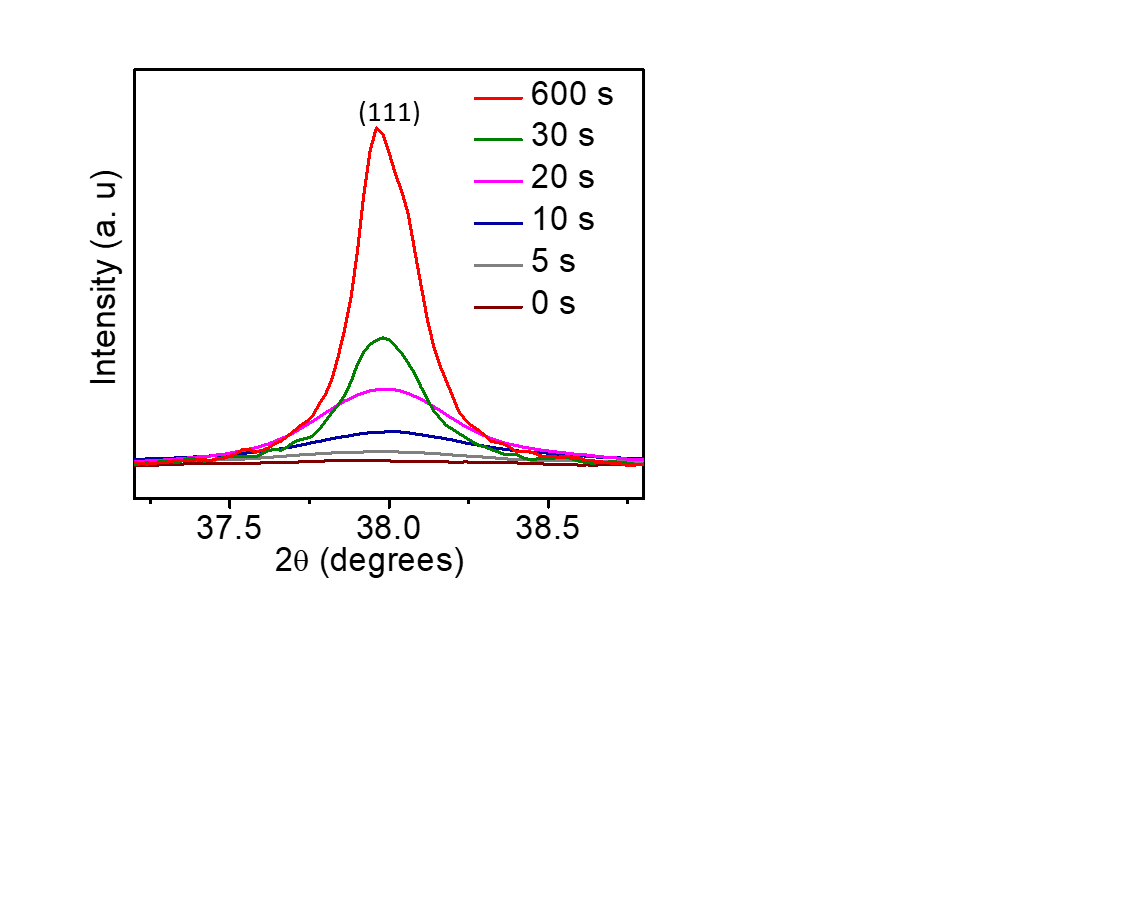


**Fig. S12** Emergence of Ag (111) peak with annealing time.

The emergence of peaks corresponding to polycrystalline Ag with annealing time formed by the ink decomposition was confirmed by XRD (Fig. 3(k)). With the annealing time, the (111) Ag peaks appear to be more prominent as shown above, indicating the conversion into purely metallic Ag film from the metal-organic complex. Notably, the peaks are still broader due to the nanometric crystallite size. However, with the increase in annealing time, the crystallite size (calculated from the Scherrer formula) increases (see Fig. 3(j)), further supporting the nucleation and growth mechanism. The average particle sizes with annealing time were also obtained from the SEM images (Figs. 3(d-i)) using the ImageJ software. However, the particle sizes are larger than the crystallite sizes, clearly indicating the growth of Ag particles with dewetting time (Fig. 3(j)).


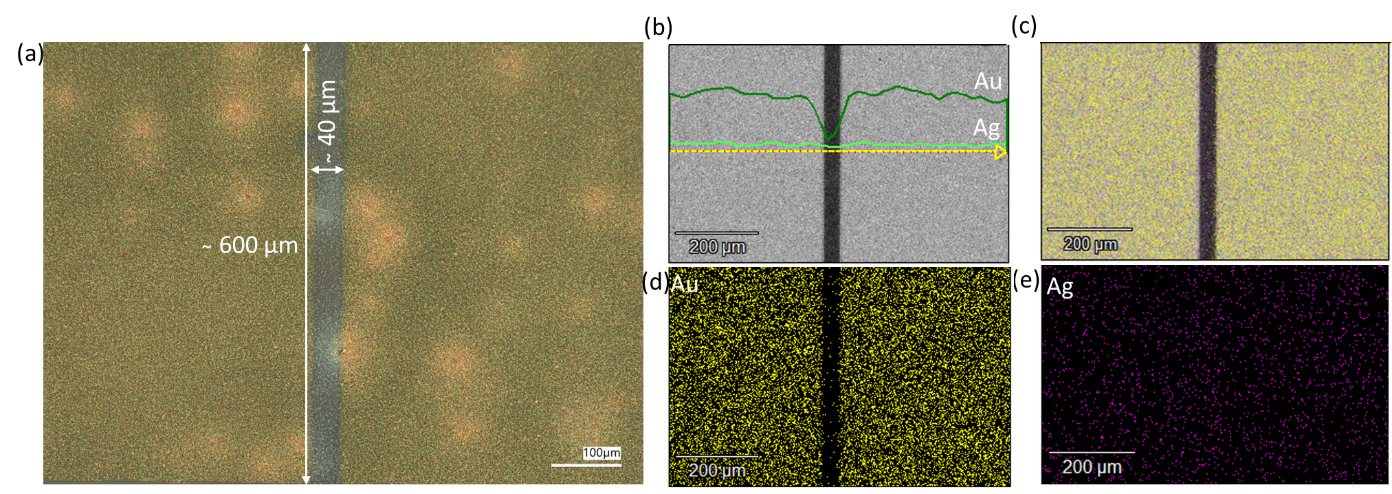


**Fig. S13** **(a)** Optical microscope image of the device indicating the device dimension. **(b)** SEM image of the device area with the line scan of Ag and Au in the inset. Color map of the device for **(c)** Ag and Au. **(d)** Au. **(e)** Ag.


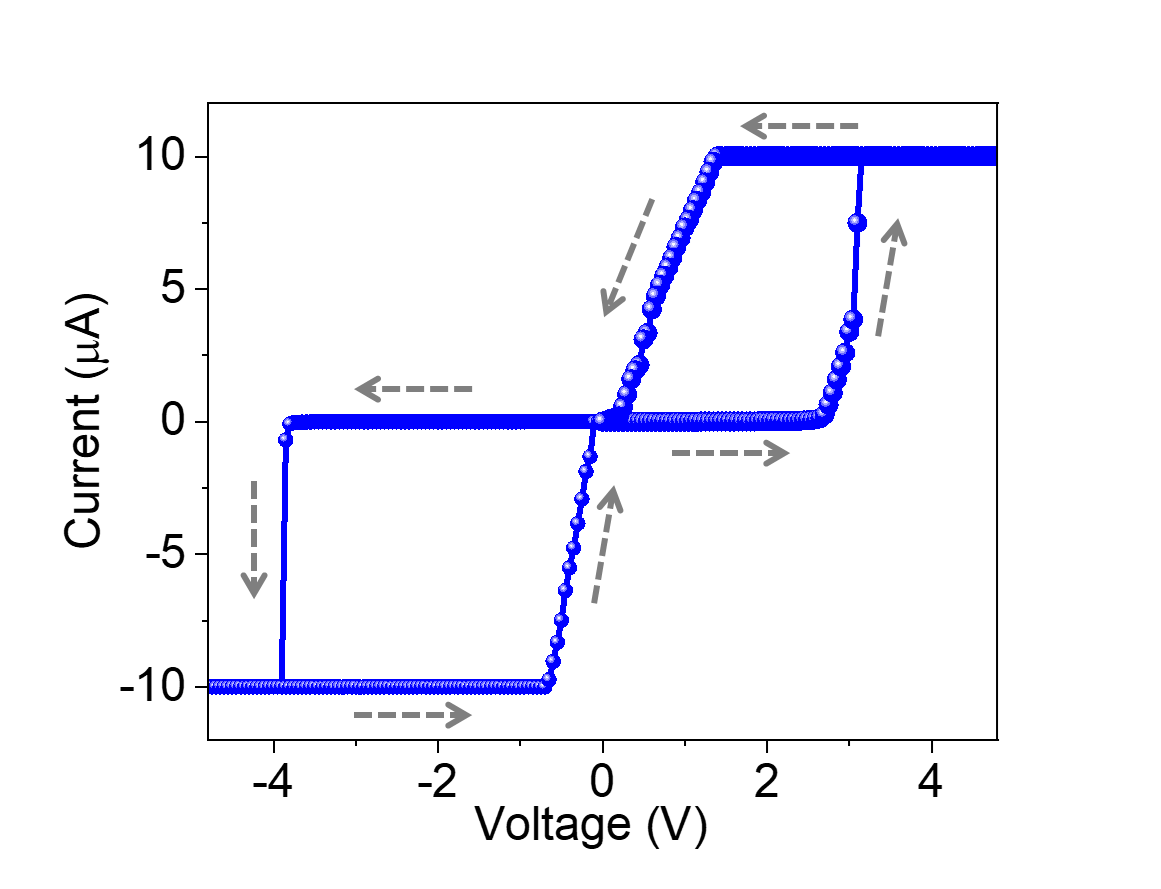


**Fig. S14** I-V characteristic of the device D6 for positive and negative sweep voltages.


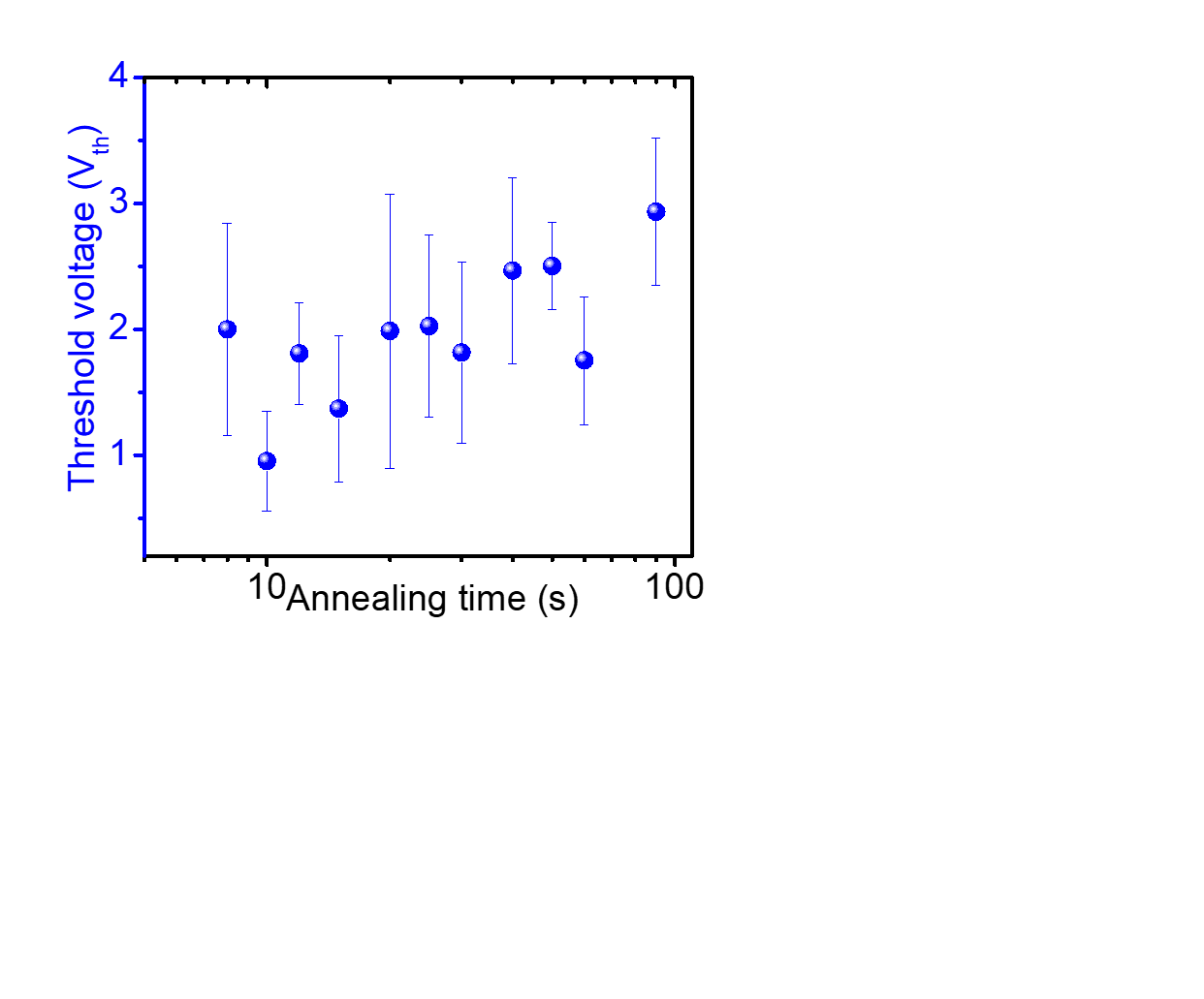


**Fig. S15** Variation of V_th_ with the annealing time.

**Relation between elastance and threshold voltage**


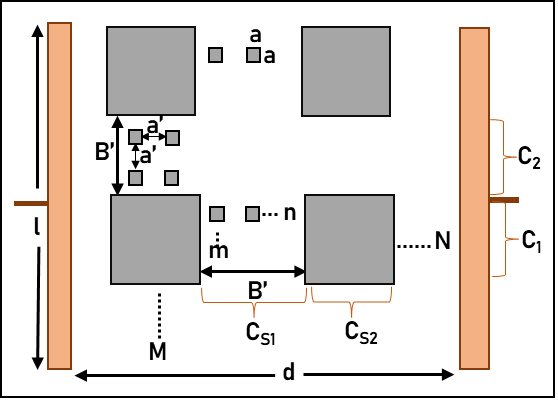


In the capacitance model, the complete device was considered to be a parallel plate capacitor with air as the dielectric and the air gap filled with Ag nanostructures (islands and particles). The dewetted Ag islands and particles are assumed to be bigger and smaller squares, respectively. The parameters considered for the capacitance calculations are as follows:

$a$is the small particle length

$a^{'}$ is the distance between small particles

$B$ is the big particle length

$B^{'}$is the distance between big particles

$M$ is the number of big particles parallel to the electrodes

$N$ is the number of big particles perpendicular to the electrodes

$m$ is the number of small particles parallel to the electrodes

$n$ is the number of small particles perpendicular to the electrodes

With the above assumptions and parameters in consideration, the capacitance is calculated for the dewetted Ag region which is found to be proportional to the V_th_. The details of the theoretical calculations are given below:

The capacitances $C_{S1}$, $C_{S2}$, $C_{1}$, and $C_{2}$ are calculated by considering the Ag nanostructures to be arranged in series and parallel to the parallel plates (in this case, Au electrodes) with air as the dielectric. Accordingly,

Capacitance $C_{S1}$ is given by;

$C_{S1}= \frac{\epsilon_{0}tm(a^{'}+a)}{n(a^{'}+a)} \left[ 1+\left( \frac{ma}{m(a^{'}+a)} \right) \left( \frac{n(a^{'}+a)}{na^{'}}-1 \right) \right]$ …S1

t is the thickness of the electrode

Further simplifying equation S1, we get;

$C_{S1}= \frac{\epsilon_{0}tm}{n} \left[ 1+\left( \frac{a^{'2}}{B(a^{'}+B)} \right) \right]$ …S2

Now, $B^{'}=n\left( a^{'}+a \right)$ and $B=m(a^{'}+a)$, this implies $\frac{m}{n}=\frac{B}{B^{'}}$.

Substituting $\frac{m}{n}$ in equation S2, we get;

$C_{S1}= \frac{\epsilon_{0}tB}{B^{'}} \left[ 1+\left( \frac{a^{2}}{a^{'}(a+a^{'})} \right) \right]$ …S3

The equation S3 was used to calculate the capacitance in the case of devices exhibiting nearly unimodal distribution.

Capacitance $C_{S2}$ is,

$C_{S2}= \frac{{k\epsilon}_{0}tB^{'}}{B}$ …S4

However, for a metal k=∞, therefore, $\frac{1}{C_{S2}}=0$

Capacitance $C_{1}$ has $n^{'}$ number of $C_{S1}$ and $C_{S2}$ in series with each other, therefore,

$\frac{1}{C_{1}}= \left( \frac{1}{C_{S1}}+ \frac{1}{C_{S2}} \right)N$ …S5

Substituting for $C_{S1}$ and $C_{S2}$ in equation S5, and replacing $N$ with $\frac{d}{b+b^{'}}$ as $d= N(b+b^{'})$

Capacitance $C_{1}$ will become.

$C_{1}= \left( \frac{B+B^{'}}{d} \right)\left[ \left( \frac{\epsilon_{0}tB}{B^{'}} \right) \left( 1+\left( \frac{a^{2}}{a^{'}(a^{'}+a)} \right) \right) \right]$ …S6

Capacitance $C_{2}$ is the again same as $C_{S1}$, therefore,

$C_{2}= \frac{\epsilon_{0}tB^{'}}{d} \left[ 1+\left( \frac{Ma}{M(a+a^{'})} \right)\left( \frac{d}{Na^{'}}-1 \right) \right]$ …S7

Substituting $d= N(a^{'}+a)$ and simplifying equation S7, we get;

$C_{2}= \frac{\epsilon_{0}tB^{'}}{d} \left[ 1+\left( \frac{a^{2}}{a^{'}(a^{'}+a)} \right) \right]$ …S8

The total capacitance $C$ is given by;

$C= M(C_{1}+C_{2})$ …S9

With $M= \frac{l}{\left( b+b^{'} \right)}$

Substituting equation S6 and S8 in equation S9, we get;

$C= \frac{\epsilon_{0}tl}{d}\left( 1+ \frac{a^{2}}{a^{'}(a^{'}+a)} \right)\left( 1+ \frac{B^{2}}{B^{'}(B^{'}+B)} \right)$ …S10

Substituting $C_{0}= \frac{\epsilon_{0}tl}{d}$ in equation S10, we get:

$C= C_{0}\left( 1+ \frac{a^{2}}{a^{'}(a^{'}+a)} \right)\left( 1+ \frac{B^{2}}{B^{'}(B^{'}+B)} \right)$ …S11

Let us consider $\left( 1+ \frac{a^{2}}{a^{'}(a^{'}+a)} \right)$ as term ‘A’ and $\left( 1+ \frac{B^{2}}{B^{'}(B^{'}+B)} \right)$ as term ‘$B$’

Now capacitance ‘$C$’ in equation S11 can be written as;

$C= \frac{\epsilon_{0}tl}{d}AB$ …S12

We know that electric field is given by;

$E= \frac{V}{d}$ …S13

$C= \frac{\epsilon_{0}a}{d}$ …S14

From equation S14 it can be inferred that;

$C \alpha\frac{1}{d}$ …S15

For electromigration to occur which is the expected phenomenon through which the Ag filaments are being formed, the electric field should be above a critical value which will be a constant for a given material and the corresponding voltage V_th_.

Now, from the equation S13, it can be inferred that.

$V_{th} \alpha d$ …S16

Combining equation S15 and S16, we get;

$V_{th} \alpha\frac{1}{C}$ …S17

$V_{th} \alpha S$ …S18

$S$ is called the electrical elastance which is the inverse of capacitance.

From equation S18, it can be inferred that the V_th_­ of the devices obtained by the I-V characteristics will be proportional to the elastance value.

Incorporating the values of $a{, a}^{'}$, $b$ and $b^{'}$ in equation S11 from the experimentally obtained dewetted parameters with the assumption of Ag islands to be the big particles and Ag particles to be the small particles, we get the capacitance values for devices D4 to D17. Considering the small particles to be squares is considerate given that most of the particles appear to be regularly shaped (see Figs. 2, S4, and S6). Though the big particles are more irregularly shaped, since their areas are narrowly distributed (see Fig. 2), they can be considered squares as well. The inverse of the capacitance value gives the theoretical elastance value which will follow a similar trend as exhibited by the V_th_ values obtained experimentally.

Thus, the theoretically obtained elastance will help us predict the V_th_ of the device and its electrical characteristics only by quantifying the dewetting patterns without carrying out the electrical measurements.

**Fill factor calculation**

Fill factor (FF) calculations were done using the ImageJ software.

The formula used for calculating the FF was:

$$FF= \frac{Area occupied by the Ag islands and particles}{Total area}(\%)$$

SEM images of active area of the devices were considered and the above formula was used to calculate the FF.


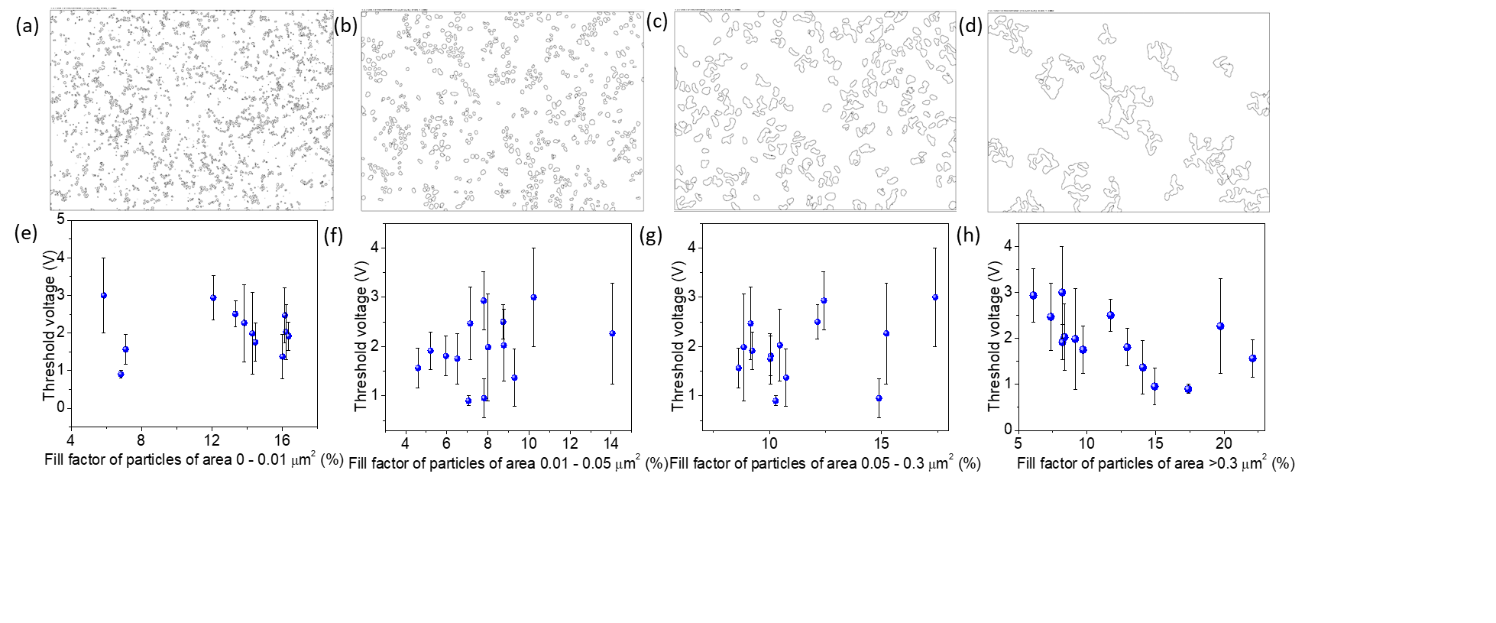


**Fig. S16** The particles are classified into areas **(a)** 0 – 0.01 µm^2^ **(b)** 0.01 – 0.05 µm^2^ **(c)** 0.05 – 0.3 µm^2^ **(d)** > 0.3 µm^2^. **(e-h)** Variation of V_th_ with FF of particles with different areas.


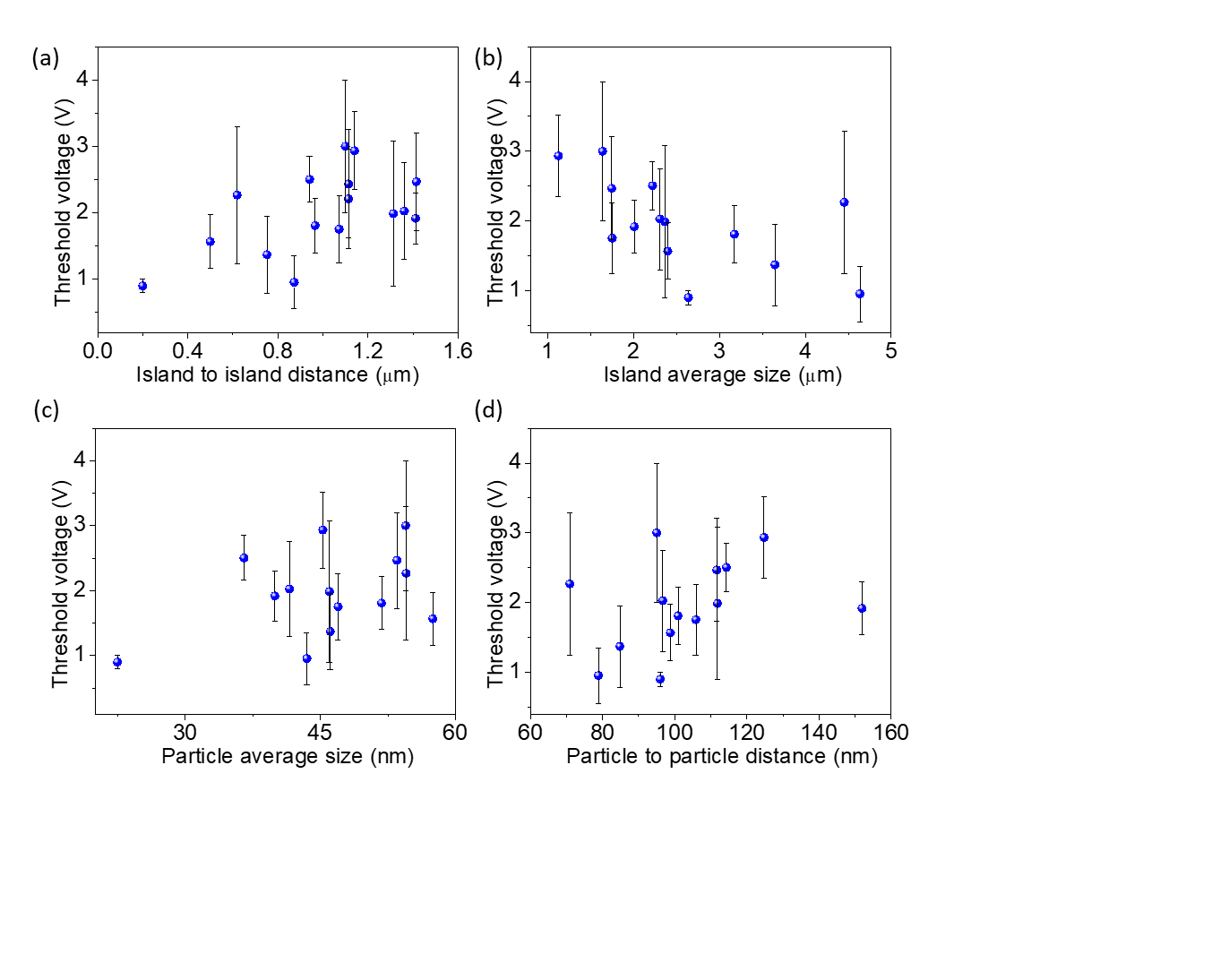


**Fig. S17** The particles of the area above > 0.3 µm^2^ are considered islands and others as particles for simplicity. Variation of V_th_ with **(a)** island to island distance $(b^{'})$ **(b)** island average size $(b)$ **(c)** particle average size $(a)$ **(d)** particle to particle distance $(a^{'})$. The values of the above parameters ($b$, $b^{'}$, $a$, and $a^{'}$) were incorporated to calculate the elastance values.

All these parameters were calculated using the ImageJ software. The island and particle average sizes were obtained by approximating them to squares.


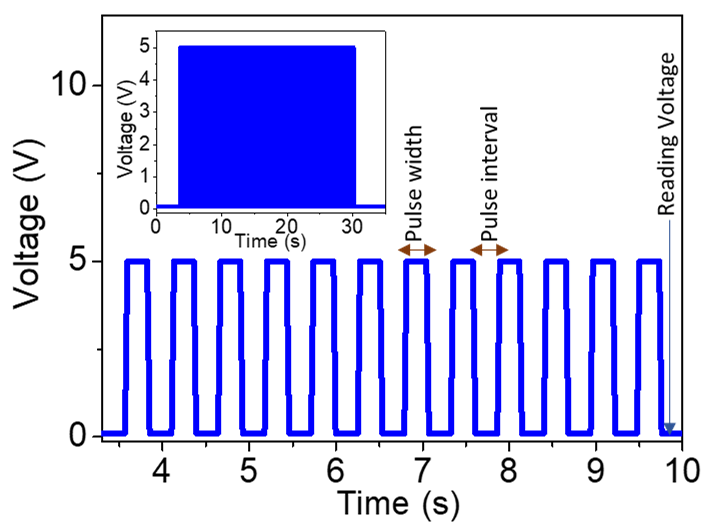


**Fig. S18** Pulse configuration with the inset showing 50 pulses of 5 V amplitude, 200 ms pulse width, and interval. The magnified image of 12 pulses from the 50 pulses clearly represents the pulsing sequence defining the pulse width, pulse interval, and the reading voltage.


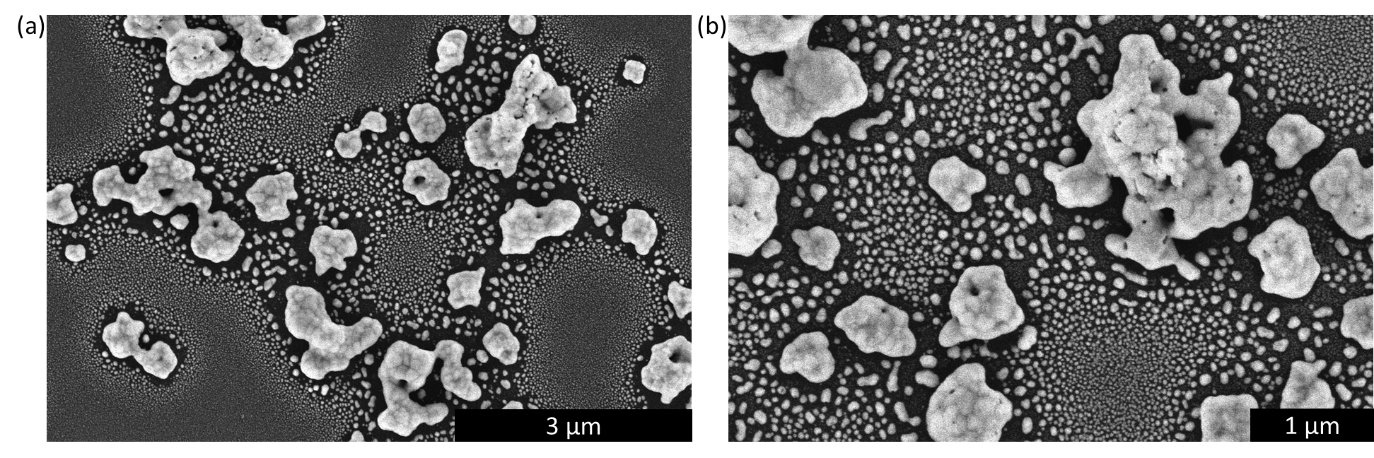


**Fig. S19** **(a)** SEM image and **(b)** magnified SEM image of the dewetted film on the large area (~ 10×10 cm^2^) substrate.
